# Natural Product-Derived Carbon Dots in Neurodegenerative Diseases: Advances in Blood–Brain-Barrier-Related Delivery, Neuroprotection, and Theranostics

**DOI:** 10.3390/biology15151235

**Published:** 2026-07-25

**Authors:** Kaixin Song, Xiang Gu, Na Sun, Rujia Xie, Ziyan Chen, Zili Wang, Ya Li, Lei Meng

**Affiliations:** 1 School of Pharmacy, Hunan University of Chinese Medicine, Changsha 410208, China; skx759959@stu.hnucm.edu.cn (K.S.); 202204010342@stu.hnucm.edu.cn (X.G.); 15874004402@163.com (R.X.); chenziy_16@stu.hnucm.edu.cn (Z.C.); 2 School of Integrated Traditional Chinese and Western Medicine, Hunan University of Chinese Medicine, Changsha 410208, China; sunna@ncst.edu.cn; 3 School of Basic Medical Sciences, North China University of Science and Technology, Tangshan 063210, China; 4 Hunan Drug Evaluation and Adverse Reaction Monitoring Center, Changsha 410013, China; zilichangsha@163.com

**Keywords:** neurodegenerative diseases, natural product-derived carbon dots, blood–brain barrier, brain delivery, neuroprotection, theranostics

## Abstract

The mechanisms underlying neurodegenerative diseases (NDDs) involve multiple pathological processes, such as abnormal protein aggregation, oxidative stress, neuroinflammation, mitochondrial dysfunction, and the disruption of neurovascular unit homeostasis. The blood–brain barrier (BBB) restricts drug exposure in the brain, posing a significant challenge for central nervous system delivery and for improving therapeutic efficacy. In recent years, carbon dots derived from natural products (CDs) have emerged as candidate materials for brain delivery and theranostic applications due to their small size, modifiable surfaces, fluorescence-tracking capability, and potential neuroprotective activity. This narrative review summarizes their sources, physicochemical characteristics, biological basis, interactions with the BBB, delivery strategies, neuroprotective effects, and imaging applications. Current evidence suggests that these CDs can alleviate oxidative stress and inflammatory responses, influence abnormal protein aggregation, and support drug delivery and fluorescence tracking in certain cellular and animal models. However, BBB permeability, brain fluorescence signals, brain parenchymal exposure, and therapeutic efficacy represent distinct levels of evidence and should not be considered interchangeable. Future studies should focus on strengthening material standardization, ensuring batch-to-batch consistency, characterizing absorption, distribution, metabolism, and excretion (ADME), conducting long-term safety assessments, and validating using humanized BBB models.

## Simple Summary

Neurodegenerative diseases gradually damage nerve cells in the brain, leading to a decline in memory, thinking, and movement. Because a protective barrier separates the blood from the brain, many medicines cannot easily enter brain tissue or reach effective levels, making these diseases difficult to treat. Natural product-derived carbon dots are very small, carbon-based materials derived from plants or other natural substances. They can be chemically modified to emit fluorescence for tracking purposes and may help to protect nerve cells. This review summarizes current research on their use to deliver treatments to the brain, reduce oxidative damage and inflammation, interfere with harmful protein clumping, and support imaging and monitoring. Studies in cells and animals suggest that some of these materials show promise. However, there is still insufficient evidence regarding how much of the material enters brain tissue, its safety during long-term use, consistency between production batches, and applicability to humans. More rigorous quality control, long-term safety testing, and experimental models that more accurately reflect the human body are required before these materials can be considered safe and reliable for diagnosing and treating brain diseases.

## 1. Introduction

Neurodegenerative diseases (NDDs) are a group of chronic disorders characterized primarily by the progressive dysfunction and loss of specific neuronal populations. The major NDDs include Alzheimer’s disease (AD), Parkinson’s disease (PD), Huntington’s disease and amyotrophic lateral sclerosis (ALS) [1,2,3,4,5]. While these diseases differ in the abnormal proteins involved, affected brain regions, and clinical manifestations, they share pathological processes such as protein misfolding and aggregation, oxidative stress, neuroinflammation, mitochondrial dysfunction, synaptic damage, and neuronal death [6,7,8,9,10,11]. As populations are aging faster than ever before, NDDs have become a leading cause of cognitive and motor impairment, as well as a significant long-term care burden [12,13,14,15]. Treating them presents the dual challenge of complex pathological networks and insufficient effective exposure within the brain. Abnormal protein aggregation, oxidative damage, chronic inflammation, disrupted energy metabolism, and neurovascular unit dysfunction interact, making single-target interventions generally insufficient to address the multiple pathological processes involved in disease progression [16,17,18]. Meanwhile, the highly selective blood–brain barrier (BBB) restricts the entry of most therapeutic molecules into the central nervous system (CNS), making it difficult to achieve adequate and sustained drug exposure in affected regions [19,20]. Consequently, material-based systems capable of combining brain delivery with multimodal therapeutic intervention have attracted considerable attention [21,22].

Carbon dots (CDs) are fluorescent nanomaterials characterized by carbon-based nanostructures and tunable surface chemistry [23]. In this review, ‘CDs’ is used as an umbrella term encompassing carbon quantum dots (CQDs), graphene quantum dots, carbonized polymer dots, and related materials. When the structure or nomenclature of a material is explicitly defined in the original study, the specific designation is retained. The size, surface chemistry, and charge characteristics of CDs can influence their interactions with biological interfaces and the BBB, thereby conferring potential for delivery, tracking, and imaging applications [24,25]. However, small particle size does not necessarily indicate effective BBB penetration, and fluorescence signals detected in brain tissue do not demonstrate that the material has entered the brain parenchyma or achieved therapeutically relevant exposure in disease-associated regions [26,27]. Natural product-derived CDs can be prepared from medicinal plants, naturally occurring bioactive molecules, and other natural raw materials. Nevertheless, natural origin does not guarantee a well-defined composition, a favorable safety profile, or clinical translatability of the final material. Carbonization, post-processing, and purification can alter the material’s composition and complicate the attribution of biological effects. Therefore, the structure and function of the resulting CDs cannot be inferred solely from the properties of their precursors [28,29,30,31,32]. Current studies have predominantly focused on AD-related cellular and animal models, suggesting that certain natural product-derived CDs may exert antioxidant and anti-inflammatory effects, modulate abnormal protein aggregation, and provide neuroprotection [33,34,35]. Nevertheless, evidence regarding BBB transport, brain parenchymal exposure, pharmacokinetics, and long-term safety remains insufficient. BBB permeability, brain fluorescence signals, brain parenchymal exposure, and therapeutic benefit represent distinct levels of evidence and should not be considered interchangeable [32,36,37,38,39].

Accordingly, this critical narrative review focuses on BBB-related delivery, neuroprotection, imaging applications, and the challenges of clinical translation associated with natural product-derived CDs in NDDs. Particular emphasis is placed on studies of natural product-derived CDs that provide material characterization and direct experimental evidence relating to NDDs, the BBB, or brain delivery. Other naturally derived materials, non-NDD models, model-protein studies, and composite nanosystems are included only as supplementary mechanistic or methodological references. The available evidence is critically evaluated with respect to material sources and characteristics, interactions with the BBB, brain exposure, functional outcomes, and translational feasibility. Particular attention is given to distinguishing brain-associated distribution, imaging capability, brain parenchymal exposure, and therapeutic effects while also considering key translational factors such as pharmacokinetics, long-term safety, material consistency, and manufacturability.

## 2. Pathophysiological Mechanisms and Therapeutic Challenges of Neurodegenerative Diseases

### 2.1. Common Pathological Mechanisms of Neurodegenerative Diseases and Their Therapeutic Implications

NDDs are a heterogeneous group of disorders characterized by neuronal dysfunction, progressive neuronal loss, and abnormal protein deposition. While individual diseases differ in their pathological proteins, selectively vulnerable brain regions, and clinical manifestations, they commonly progress through interconnected pathological processes, including abnormal protein aggregation, oxidative stress, mitochondrial dysfunction, chronic neuroinflammation, disruption of neurovascular unit (NVU) homeostasis, and synaptic injury [17,40,41,42,43,44,45]. 

The core pathological features of AD include amyloid-β (Aβ) deposition and abnormal phosphorylation and aggregation of tau protein. These alterations may be accompanied by glial activation, oxidative stress, neuroinflammation, and BBB dysfunction, which collectively promote synaptic injury and neurodegeneration [46,47,48,49,50,51]. PD is primarily characterized by progressive loss of dopaminergic neurons in the substantia nigra and abnormal aggregation of α-synuclein (α-syn). Misfolding and aberrant modification of α-syn can promote the formation of toxic aggregates and Lewy bodies and, together with mitochondrial dysfunction, oxidative stress, and glial activation, can exacerbate neuronal injury [52,53,54]. Other NDDs may involve mutant huntingtin, TAR DNA-binding protein 43 (TDP-43), fused in sarcoma (FUS), or other pathological proteins, and are often associated with proteostasis disruption, cellular stress, and selective neuronal vulnerability [10,55,56,57,58,59].

Protein misfolding, aggregation, and impaired clearance are major shared mechanisms across multiple NDDs. The persistent accumulation of pathological proteins, including Aβ, tau, α-syn and TDP-43, can disrupt proteostasis and impair mitochondrial function, membrane trafficking and synaptic function. Accordingly, inhibiting abnormal protein aggregation and promoting protein clearance represent potential disease-modifying strategies [60,61,62,63,64]. Additionally, oxidative stress, mitochondrial dysfunction, and chronic neuroinflammation can reinforce each other to form self-amplifying pathological loops that further aggravate synaptic and neuronal injury. Therefore, strategies that restore redox homeostasis, preserve mitochondrial function, and alleviate neuroinflammation are also important for neuroprotection [65,66]. These interconnected pathological processes provide the principal biological rationale for investigating the anti-oxidative, anti-inflammatory, anti-aggregation, and neuroprotective effects of CDs derived from natural products.

**Figure 1:**
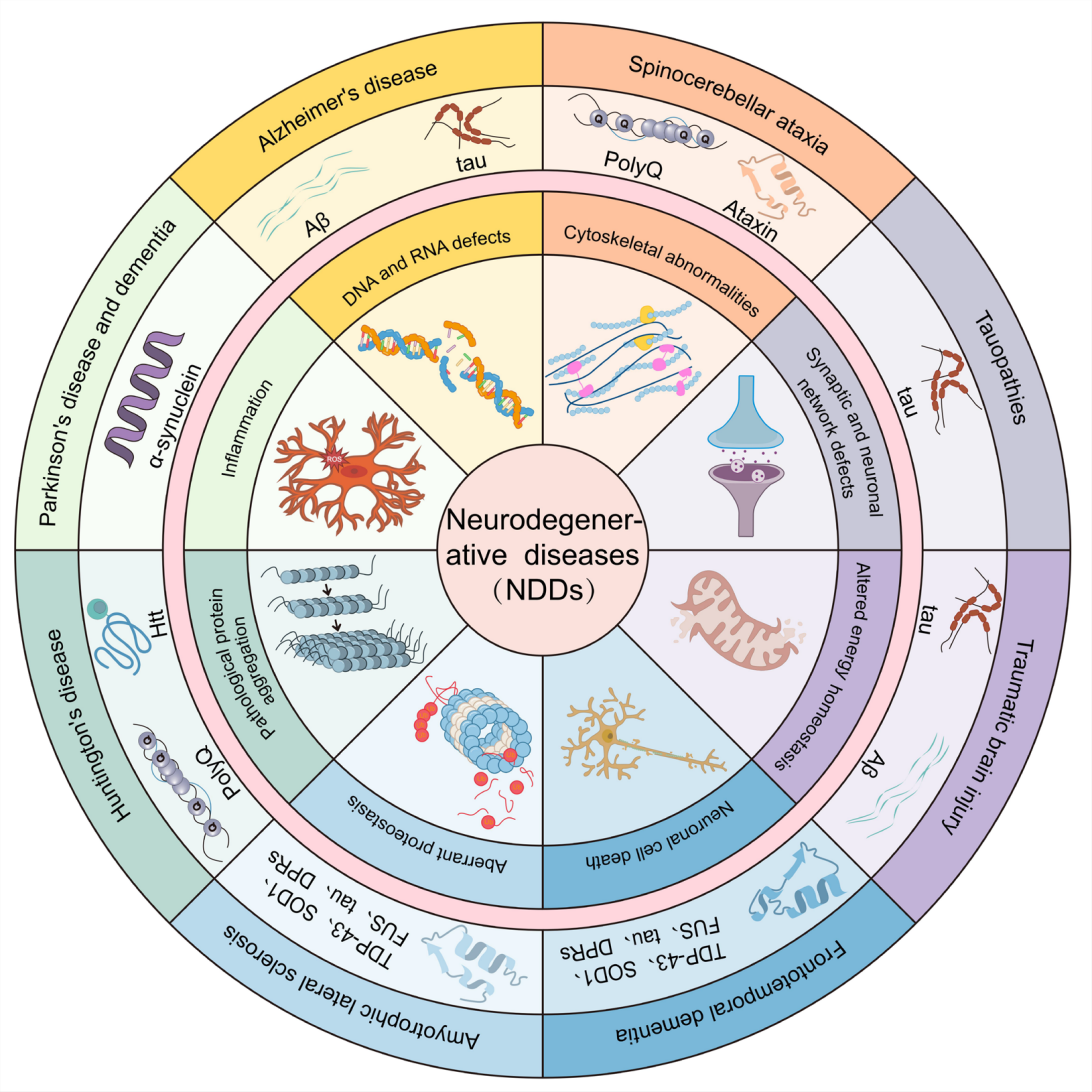
Shared pathological mechanisms and representative proteinopathies across major neurodegenerative diseases. Different NDDs are associated with abnormal proteins and brain regions that are relatively specific to each disease. However, their pathological progression often involves interconnected processes, including abnormal protein aggregation, neuroinflammation, mitochondrial dysfunction, synaptic impairment, and neuronal loss. Colors are used only to distinguish different disease categories, pathological proteins, and pathological mechanisms and do not represent quantitative differences. Abbreviation: NDDs, neurodegenerative diseases.

Table 1 summarizes the representative abnormal proteins, the principal affected brain regions, the characteristic pathological features, and the clinical manifestations of the major NDDs, and Figure 1 provides an overview of the associated proteinopathies and their common pathological mechanisms.

### 2.2. Limitations of Current Treatments and Unmet Needs

NDDs are characterized by prolonged disease courses and complex pathogenic mechanisms. Current treatments primarily aim to relieve symptoms or slow disease progression, but face challenges including insufficient exposure of therapeutic molecules to the brain, limitations of single-target interventions, long-term safety concerns, and poor treatment adherence [87,88]. The BBB restricts the entry of many therapeutic molecules into the CNS, making it difficult to achieve adequate and sustained drug exposure in disease-relevant brain regions [89,90,91]. Furthermore, detecting a drug or material in brain tissue does not necessarily indicate that an effective concentration has been reached at the pathological site. Therefore, brain exposure should be evaluated comprehensively alongside pathological localization and functional outcomes [92,93]. As multiple pathological processes interact, interventions targeting a single molecule or pathway are generally insufficient to address the complex networks underlying disease progression. Therapeutic evaluation should also incorporate neuroprotective effects and disease-relevant functional outcomes [17,45,94].

NDDs often require long-term or repeated treatment, and treatment sustainability may be affected by adverse effects, the burden of administration, and patient adherence. Increasing the dose when brain exposure is insufficient may enhance peripheral exposure and systemic toxicity without necessarily producing a corresponding therapeutic benefit in the CNS [95,96,97]. For nanosystems designed for brain delivery, attention should also be given to the potential neurotoxicity and inflammatory responses associated with long-term material accumulation [98]. Therefore, emerging therapeutic systems for NDDs must improve effective brain exposure, address multiple pathological processes, and ensure safety during long-term use.

### 2.3. Impact of Blood–Brain Barrier and Neurovascular Unit Dysfunction on Treatment

The BBB is a key interface that maintains homeostasis within the CNS. Primarily composed of brain microvascular endothelial cells and their tight junctions, it also includes pericytes, the basement membrane, astrocytic endfeet, and neurons, collectively constituting the NVU [99,100]. Under physiological conditions, the BBB restricts the entry of foreign substances into the brain parenchyma, thereby preserving CNS homeostasis via tight junctions, low transcytosis rates, and selective transport and efflux systems [101,102].

In NDDs, however, the BBB/NVU can undergo structural and functional alterations, including impaired endothelial barrier function, disrupted tight junctions, reduced pericyte support, and altered glial reactivity [103,104,105]. These changes can compromise barrier integrity and disrupt the brain microenvironment, allowing harmful components and inflammatory signals in the blood to abnormally enter brain tissue, thereby aggravating neuroinflammation, synaptic dysfunction, and neuronal injury [105,106]. Therefore, BBB/NVU dysfunction not only affects the brain distribution of therapeutics but also represents an important pathological process that contributes to disease progression.

It is important to note that enhancing a therapeutic system’s ability to cross the BBB and repairing pathological BBB damage are distinct objectives. The former aims to improve effective brain exposure while preserving the barrier’s selective properties, whereas the latter focuses on restoring barrier integrity and brain homeostasis [107,108]. Pathological increases in BBB permeability are commonly accompanied by vascular inflammation and barrier injury and should not be interpreted solely as improved delivery efficiency [105]. Therefore, when evaluating brain-delivery systems such as natural product-derived CDs, assessments should extend beyond brain distribution to include effective brain exposure, localization within pathological regions, BBB/NVU integrity, and disease-relevant functional outcomes.

Current in vitro BBB models cannot fully reproduce the structure and function of the NVU, and interspecies differences in barrier properties further limit the direct extrapolation of experimental findings to humans [109,110,111]. Evidence of brain delivery from animal studies should therefore be evaluated hierarchically, alongside brain parenchymal exposure and localization within pathological regions. Whole-brain fluorescence or total tissue content alone is insufficient to demonstrate that a material has effectively crossed the BBB [112,113].

## 3. Natural Product-Derived Carbon Dots: Physicochemical Basis, Blood–Brain-Barrier-Related Mechanisms, and Evidence Boundaries

### 3.1. Physicochemical Characteristics and Biological Basis of Natural Product-Derived Carbon Dots

CDs derived from natural products have attracted increasing attention in nanomedicine due to their nanoscale dimensions, tunable surface chemistry, fluorescence properties, and potential bioactivity [25,28,114]. These CDs are typically smaller than 10 nm [115]. Their small size may facilitate dispersion, cellular uptake and tissue diffusion, while their carbon core structures, surface states and heteroatom doping may confer fluorescence imaging capability and potential biological activity [116,117,118]. However, effective brain delivery requires more than just small particle size and fluorescence; their in vivo behavior is also influenced by factors such as surface chemistry, protein corona formation, route of administration, and the status of biological barriers [113,119].

The surfaces of natural product-derived CDs commonly contain functional groups, such as hydroxyl, carboxyl, and amino groups. These groups can improve water solubility and chemical modifiability and provide reactive sites for the conjugation of drugs or targeting ligands [120,121]. Defects in the carbon core, surface functional groups, and heteroatom doping may contribute to free-radical scavenging, enabling certain CDs to exhibit antioxidant and neuroprotective enzyme-like activity [122,123]. Nevertheless, most current studies rely primarily on conventional physicochemical and spectroscopic characterization. The relationships between structural heterogeneity, surface composition, defect states and the optical and biological effects of CDs are not fully understood, and their structure–property–activity relationships remain to be elucidated [124,125].

Natural product-derived CDs are commonly prepared from medicinal plants, food-derived materials, naturally occurring bioactive small molecules, or biomacromolecules using hydrothermal, solvothermal, or pyrolytic methods [126,127,128]. However, a natural origin does not necessarily indicate low toxicity, retention of the precursor’s pharmacological activity, or batch-to-batch stability. The composition of natural precursors can vary with geographical origin, harvest period, and extraction method, and carbonization can substantially alter their original constituents. Inadequate purification may also leave residual precursors or molecular by-products that interfere with material characterization and the attribution of biological effects [39,129,130,131]. Therefore, studies of natural product-derived CDs should be based on clearly defined material identity, adequate purification, consistent batch-to-batch performance, and well-established structure–function relationships.

**Figure 2:**
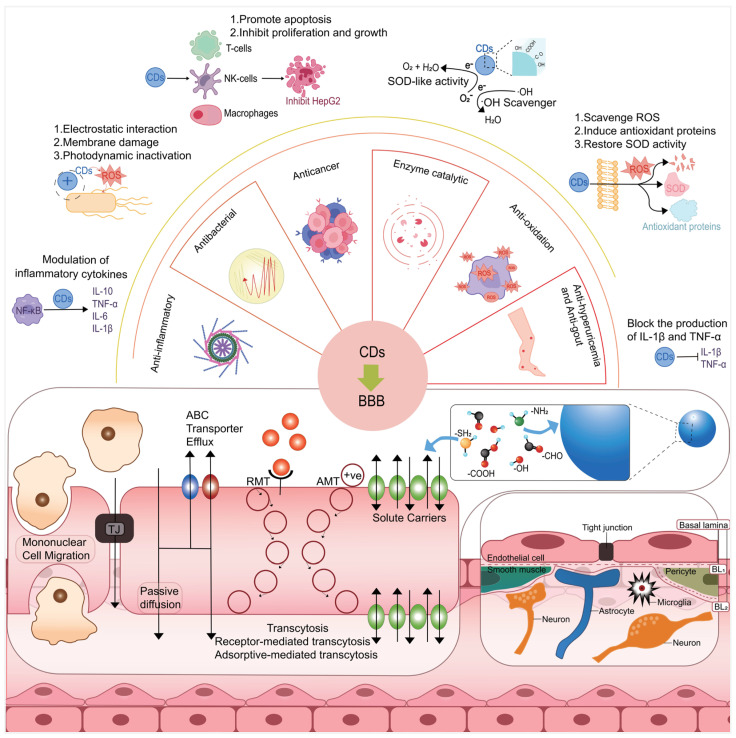
Schematic illustration of the multifunctional bioactivities of natural product-derived carbon dots and their potential mechanisms of interaction with the blood–brain barrier. The figure summarizes the major biological activities of natural product-derived CDs and the potential mechanisms underlying their interactions with the BBB. These activities and transport pathways depend on the specific material and experimental model, and some mechanisms have been validated only in particular CD systems. Arrows indicate the proposed directions of transport or functional regulation, whereas colors distinguish different functional and mechanistic modules and do not indicate quantitative differences. Abbreviations: ABC, ATP-binding cassette; AMT, adsorptive-mediated transcytosis; BBB, blood–brain barrier; CDs, carbon dots; RMT, receptor-mediated transcytosis; ROS, reactive oxygen species; SOD, superoxide dismutase; TJ, tight junction.

Table 2 summarizes the precursor categories, representative structural features, and reported biological functions of natural product-derived CDs, and Figure 2 depicts their multifunctional bioactivities and potential BBB-related transport mechanisms.

### 3.2. Interactions of Natural Product-Derived Carbon Dots with the Blood–Brain Barrier and Potential Transport Mechanisms

The potential applications of CDs derived from natural products in NDDs are closely associated with their interactions with the BBB and their distribution within the brain [24]. Their water solubility, colloidal stability, protein corona formation, and interactions with brain microvascular endothelial cells can be influenced by particle size, surface charge, and surface functional groups, including hydroxyl, carboxyl, and amino groups [102,132,157,158,159,160]. This affects cellular uptake and in vivo distribution. Some natural product-derived CDs have been reported to exhibit brain-associated distribution or interactions with the BBB. However, small size, endothelial uptake, or signals detected in brain tissue do not directly demonstrate quantifiable and reproducible exposure within the brain parenchyma [112,113,161].

Receptor- or carrier-mediated transport provides a mechanistic basis for designing CD-based brain delivery systems. Transferrin-modified CDs may enhance interactions with the transferrin receptor [157], whereas receptor-mediated pathways involving the lactoferrin receptor also provide a basis for the brain-targeted functionalization of CDs [162,163,164]. Tryptophan-derived CDs may be taken up via L-type amino acid transporter 1 (LAT1), while glucose- or trehalose-functionalized carbonized polymer dots may be associated with glucose transporter 1 (GLUT1)-related pathways [161,165]. Nevertheless, receptor binding, endothelial uptake, or increased permeability in vitro alone cannot demonstrate that a material has crossed an intact BBB and reached an effective concentration in the brain parenchyma. BBB injury and altered permeability under pathological conditions may also affect the brain distribution of CDs. Neuroinflammation or acute brain injury can disrupt tight junctions and increase barrier leakage, thereby facilitating the passive entry of materials. However, such distribution should not be equated with active transport across an intact BBB under physiological conditions [102,166,167].

Overall, the interactions of natural product-derived CDs with the BBB and their distribution within the brain are influenced by multiple factors, including the characteristics of the material’s surface, interactions with the biological interface, and the pathological status of the barrier. BBB interactions, in vitro permeability, brain tissue signals, brain parenchymal exposure, and therapeutic effects represent distinct levels of evidence and should not be considered interchangeable. Future studies should integrate confirmation of material identity, quantitative assessment of brain distribution and validation of transport mechanisms, establishing an evidence chain linking ‘material characteristics–brain parenchymal exposure–functional effects’.

## 4. Advances in the Application of Natural Product-Derived Carbon Dots in Neurodegenerative Diseases

**Figure 3:**
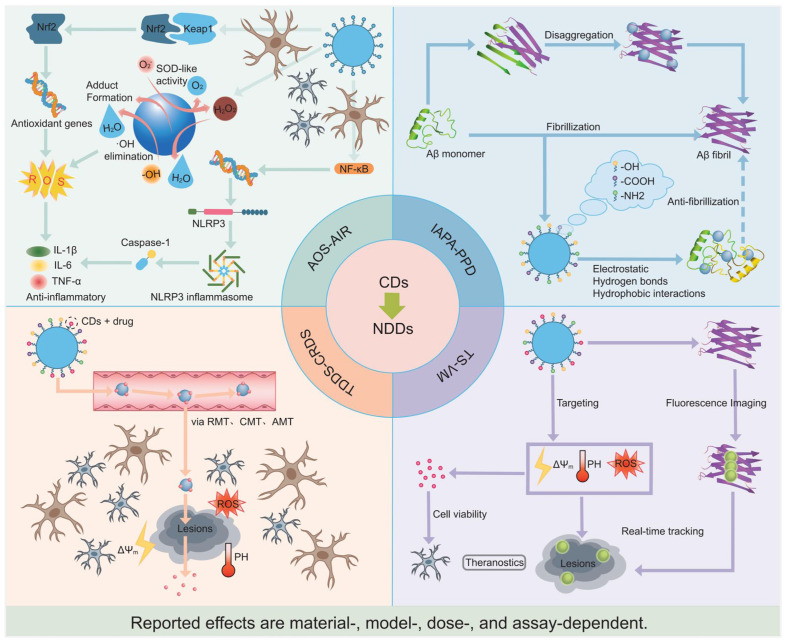
Multifunctional mechanisms and theranostic applications of natural product-derived carbon dots in neurodegenerative diseases. Schematic illustration of the multifunctional roles of CDs in NDDs. CDs perform multifunctional roles through antioxidative and anti-inflammatory regulation (AOS-AIR), inhibition of abnormal protein aggregation (IAPA-PPD), targeted drug delivery across the BBB (TDDS-CDs), and fluorescence imaging-guided theranostics (TS-IM). Arrows indicate the proposed directions of mechanistic processes, whereas colors distinguish the four functional modules and do not indicate quantitative differences.

NDDs involve multiple pathological processes, including oxidative stress, neuroinflammation, and abnormal protein aggregation. Interventions targeting a single pathway often fail to achieve sufficient therapeutic efficacy [45,168]. Natural product-derived CDs may exert neuroprotective effects by modulating oxidative stress and inflammatory responses and inhibiting abnormal protein aggregation. Their BBB-related delivery and fluorescence imaging properties also have potential applications in theranostics [33,169]. This narrative review therefore summarizes recent advances in natural product-derived CDs for NDDs, emphasizing their principal mechanisms of action and major application areas (Figure 3).

### 4.1. Antioxidant Effects and Regulation of Inflammation

Oxidative stress and chronic neuroinflammation are two major mechanisms that underlie the progression of NDDs. These processes can reinforce each other, jointly contributing to mitochondrial dysfunction, synaptic injury, and neuronal death [170,171,172]. Natural product-derived CDs have tunable carbon core structures, surface functional groups, and defect sites that may enable them to scavenge ROS, regulate redox homeostasis, and modulate inflammation-related signaling pathways [173,174]. Accordingly, natural product-derived CDs have demonstrated preclinical potential to mitigate oxidative damage and modulate the inflammatory microenvironment associated with NDDs. However, it should be noted that reductions in ROS levels or inflammatory mediators primarily reflect mechanistic effects in specific experimental models and do not directly demonstrate effective brain exposure or the ability to delay long-term disease progression [36,38,175]. This section discusses the neuroprotective potential of natural product-derived CDs from two perspectives: their antioxidative effects and their ability to modulate inflammatory responses.

#### 4.1.1. Antioxidant Effects

In NDDs such as AD and PD, excessive accumulation of ROS can induce lipid peroxidation, oxidative protein damage, mitochondrial dysfunction, and neuronal apoptosis. This contributes to neuronal injury and disease progression [172,176]. Studies have shown that some CDs derived from natural products possess free-radical-scavenging activity and can alleviate oxidative stress by reducing ROS levels. Certain CDs have also been reported to exhibit antioxidant enzyme-mimetic activity, participating in the regulation of redox homeostasis by catalytically removing ROS [172,177,178,179].

The antioxidant activity of CDs derived from natural products is closely associated with their carbon-core structures, surface functional groups, defect sites, and heteroatom doping. For instance, *Polygonatum–Gastrodia-derived* CDs may exhibit free-radical-scavenging capacity and antioxidative activity due to their oxygen-containing surface groups, defect sites, and carbon-core structures, which serve as potential active centers [180]. Heteroatom doping can also modulate the electronic structure and surface active sites of CDs, enhancing their ability to scavenge ROS and related reactive species, and improving their antioxidant activity [122].

In addition to directly scavenging ROS, natural product-derived CDs may exert protective effects by regulating intracellular antioxidant defense systems. For instance, Radix Isatidis-derived CDs have been reported to cross the BBB and reduce ROS levels, suppress neuroinflammation and Aβ aggregation, and improve AD-like pathological phenotypes in a mouse model [33]. Epigallocatechin gallate (EGCG)-derived carbonized polymer dots, curcumin-derived CDs, caffeic acid-derived CDs, and vitamin C-derived CDs have also been reported to scavenge ROS, alleviate oxidative stress, modulate abnormal protein aggregation, or reduce injury in neural cells [34,174,181,182].

Overall, the antioxidant effects of natural product-derived CDs mainly involve the direct removal of excessive ROS and the regulation of endogenous antioxidant enzyme systems. These effects may alleviate oxidative stress-related cellular injury and provide a material basis for antioxidant interventions in NDDs. However, apart from a limited number of AD-related animal studies, the available evidence largely concerns changes in ROS levels, antioxidant enzyme activity, and cellular injury markers. These findings cannot be equated directly with long-term disease-modifying effects and require further validation through assessments of brain distribution, pharmacokinetics, dose–response relationships and long-term safety.

#### 4.1.2. Regulation of Inflammatory Responses

Chronic neuroinflammation is an important underlying mechanism of the sustained progression of NDDs. Aberrant microglial activation, release of pro-inflammatory cytokines, and inflammasome activation can collectively promote neuronal injury and synaptic dysfunction. CDs derived from natural products have shown potential to modulate neuroinflammatory processes by regulating inflammation-related signaling pathways, suppressing the excessive activation of inflammatory cells and reducing the expression of pro-inflammatory mediators [183,184,185]. Previous studies have demonstrated that CDs derived from natural products or constructed using naturally occurring bioactive compounds can reduce the levels of pro-inflammatory mediators such as tumor necrosis factor-α (TNF-α), interleukin-1β (IL-1β), interleukin-6 (IL-6), and nitric oxide (NO), thereby alleviating inflammation-associated cellular injury [186,187,188]. For instance, red-fluorescent cerium-based CDs prepared using curcumin have been shown to reduce the expression of TNF-α and IL-6 in BV-2 microglial cells, indicating their capacity to suppress inflammatory responses in microglia [187]. EGCG-derived CQDs have also been reported to reduce inflammatory cytokine release and alleviate neuroinflammation-associated injury [188].

Regarding inflammatory signaling pathways, nuclear factor kappa B (NF-κB) is a key regulator of neuroinflammatory responses. Its activation can promote the assembly of the NOD-like receptor family pyrin domain-containing 3 (NLRP3) inflammasome and induce the release of inflammatory cytokines, such as IL-1β and interleukin-18 (IL-18) [189,190,191,192,193]. Accordingly, modulation of the NF-κB/NLRP3 inflammatory axis is an important strategy for alleviating neuroinflammation. Studies have demonstrated that purple sweet potato-derived CDs can inhibit the activation of the Toll-like receptor 4 (TLR4)/NF-κB/NLRP3 signaling axis in lipopolysaccharide (LPS)-induced inflammatory models. They can also downregulate pro-inflammatory cytokines, including IL-1β, IL-6, and TNF-α, and attenuate pyroptosis-related responses [186]. In an intracerebral hemorrhage model, EGCG-based carbon nanodots inhibited components of the inflammatory cascade, including NF-κB, NLRP3, caspase-1, and gasdermin D (GSDMD), reducing pro-inflammatory cytokine levels and ameliorating inflammation-associated neurological injury [188].

Overall, the anti-inflammatory effects of CDs derived from natural products are manifested in three ways: suppression of excessive activation of microglia and other inflammatory cells, reduction in pro-inflammatory mediators such as TNF-α, IL-1β, IL-6, and NO, and regulation of inflammation-related signaling pathways including the TLR4/NF-κB/NLRP3 axis. This attenuates inflammatory cascades and pyroptosis. Current studies provide preliminary experimental evidence that natural product-derived CDs can improve the neuroinflammatory microenvironment. However, most of the available evidence has been obtained from BV-2 cells, LPS-induced inflammatory models, epilepsy models, or acute brain injury models, and therefore cannot be equated directly with long-term therapeutic effects in chronic NDDs such as AD and PD. Future studies should integrate assessments of brain exposure, glial phenotypic transitions, dose–response relationships, and long-term safety to further clarify the relevance of their anti-inflammatory effects to disease and their translational value.

### 4.2. Inhibition of Abnormal Protein Aggregation and Regulation of Proteostasis

Protein misfolding, aggregation, and impaired clearance are major pathological features of NDDs [194,195]. In diseases such as AD, PD, and ALS, abnormal proteins including Aβ, tau, α-syn, and TDP-43 can form toxic oligomers and fibrillar aggregates. These aggregates subsequently induce synaptic injury, mitochondrial dysfunction, oxidative stress, and neuroinflammation [196,197,198,199]. Recent studies have shown that CDs derived from naturally occurring bioactive molecules or natural products, together with related functionalized carbon-based quantum dots, may interfere with pathological protein aggregation. This is achieved by inhibiting the initiation of aggregation and fibril elongation, promoting aggregate disassembly, reducing aggregate-associated toxicity, and regulating proteostasis [34,200].

It should be noted that these studies vary considerably in the sources of the materials used, their surface structures, and the disease models employed. Some of the evidence is derived from non-natural-product-derived or functionalized CD systems and is therefore more appropriately regarded as mechanistic evidence than as direct therapeutic evidence for natural product-derived CDs in NDDs.

#### 4.2.1. Inhibition of Protein Aggregation Initiation and Fibril Elongation

The toxicity of proteins associated with NDDs is closely linked to their misfolding, oligomer formation, and subsequent fibrillization [201,202]. Some CDs can interact with protein monomers or oligomers during the early stages of aggregation, thereby preventing further conversion into mature fibrils and inhibiting abnormal protein aggregation. For instance, sodium citrate-derived CQDs have been reported to prevent the conversion of Aβ monomers and oligomers into mature fibrils and to promote oligomer dissociation, thereby suppressing amyloid aggregation [203]. While this system is not strictly classified as a natural product-derived CD, it provides evidence that CDs can interfere with the initial stages of Aβ aggregation.

CDs derived from naturally occurring bioactive molecules have also shown potential to inhibit abnormal protein aggregation. For instance, EGCG-derived carbonized polymer dots can interact with Aβ aggregates through their aromatic hydrophobic structures and surface functional groups, such as hydroxyl and carboxyl groups. These interactions prevent the addition of further monomers to fibril ends, thereby inhibiting Aβ fibril elongation [182]. Additionally, quercetin-derived red-emissive carbon nanoparticles have been reported to inhibit Aβ aggregation and exhibit anti-amyloid fibrillization activity [204]. Taken together, these findings suggest that CDs derived from naturally occurring bioactive molecules may interfere with abnormal protein aggregation through multiple interfacial interactions, including hydrophobic, π–π, hydrogen-bonding, and electrostatic interactions.

Overall, CDs derived from naturally occurring bioactive molecules and related carbon-based nanomaterials may interfere with the initiation of abnormal protein aggregation and fibril elongation. This provides a material basis for anti-aggregation therapeutic strategies. However, the current evidence is predominantly derived from in vitro protein aggregation assays and does not directly demonstrate stable anti-aggregation effects in the complex brain microenvironment or long-term disease-modifying activity.

#### 4.2.2. Promotion of Aggregate Disassembly and Reduction in Aggregate Toxicity

In addition to inhibiting the initiation of aggregation, some CDs derived from naturally occurring bioactive molecules, as well as related functionalized CDs, can promote the disassembly of mature aggregates and reduce aggregate-associated toxicity. Amino-functionalized CQDs have been shown to interact with amyloid proteins via their surface functional groups, thereby altering the aggregation of hen egg white lysozyme and inducing the formation of less toxic spherical aggregates [205]. While this system uses functionalized CDs and the model protein is not one associated with AD or PD, it shows that modifying the surface chemistry can significantly impact the interaction between CDs and amyloid proteins.

CDs derived from naturally occurring bioactive molecules may also promote the disassembly of mature amyloid fibrils. For instance, flavonoid-derived carbonized polymer dots have been shown to inhibit the aggregation of both hen and human lysozyme amyloid proteins in a dose-dependent manner, while also promoting the degradation of mature fibrils into smaller aggregates [206]. Red-emissive quercetin-derived CDs have been reported to rapidly disassemble mature Aβ fibrils whilst also exhibiting ROS-scavenging and Aβ plaque-imaging capabilities. In an AD nematode model, these CDs reduced toxicity and prolonged lifespan, suggesting the potential integration of anti-aggregation, antioxidant, and imaging functions [204]. Additionally, plant-derived fluorescent CDs can modulate copper-induced disturbances in lipid metabolism and protein aggregation through copper-ion chelation. This suggests that they may alleviate associated neurotoxicity by interfering with the ‘metal-ion imbalance–lipid metabolic disturbance–protein aggregation’ cascade [207].

Overall, CDs derived from naturally occurring bioactive molecules may regulate multiple stages of pathological protein aggregation by disassembling mature fibrils, scavenging ROS, modulating metal-ion homeostasis, and reducing aggregate-associated cytotoxicity. However, the available findings are largely based on in vitro aggregation systems, nematode models, or short-term toxicity assessments. There is a lack of causal evidence linking these effects to brain exposure, reduced pathological protein burden, and long-term behavioral improvement.

#### 4.2.3. Regulation of Proteostasis and Reduction in Abnormal Protein Burden

The effects of CDs derived from natural products and their functionalized derivatives on pathological protein processes extend beyond inhibiting aggregation and promoting aggregate disassembly. They may also involve regulating proteostasis, restoring autophagic clearance, and reducing abnormal protein burden [127,165,195]. For instance, curcumin-derived CQDs can enhance the water solubility and bioavailability of curcumin while simultaneously reducing Aβ aggregation and tau hyperphosphorylation. This suggests a potential role in regulating AD-related proteostasis [34]. Additionally, glucose- or trehalose-functionalized carbon-based polymeric nanoparticles loaded with plasmid DNA (pDNA) can facilitate nuclear gene delivery under ROS-responsive conditions. These nanoparticles can silence the expression of *SNCA*, the gene encoding α-syn, restore autophagic flux, reduce α-syn aggregation, alleviate neuroinflammation, and improve motor function in PD mice [165]. This study suggests that CD-related delivery systems may facilitate the clearance of abnormal proteins by regulating genes and restoring autophagy.

While some CDs have not been directly demonstrated to promote abnormal protein degradation, they may contribute to proteostasis regulation by inhibiting aggregation or reducing aggregate burden. For instance, phenylalanine-derived CDs can bind to and stabilize α-syn monomers and dimers via hydrophobic interactions, thereby preventing further aggregation. They have also been reported to enhance motor function in PD mice after crossing the BBB [208]. Congo red-derived CDs can simultaneously inhibit tau and Aβ aggregation, providing experimental evidence for dual-target intervention against abnormal protein pathology in AD [209]. Graphene quantum dots (GQDs) derived from coffee waste and CDs derived from camel milk have also been reported to inhibit α-syn fibrillization, suggesting that they may reduce the burden of PD-related abnormal protein aggregates [210,211]. However, not all of these systems are strictly classified as natural product-derived CDs, and aggregation inhibition remains the primary endpoint in most studies. Therefore, these findings cannot be equated directly with enhanced protein degradation or clearance.

In summary, CDs derived from natural products or naturally occurring bioactive molecules, together with related functionalized CDs, may modulate NDD-associated protein pathology by inhibiting Aβ, tau, and α-syn aggregation, promoting the disassembly of mature fibrils, regulating metal-ion homeostasis, restoring autophagic flux, or reducing abnormal protein burden. These findings provide important directions for the design of multitarget neuroprotective and theranostic systems. However, current studies have several notable limitations. Firstly, some materials are not strictly derived from natural products, resulting in heterogeneous levels of evidence. Secondly, many studies remain limited to in vitro protein aggregation assays or short-term experimental models. Thirdly, it is unclear whether reductions in abnormal protein burden result from genuine brain exposure, direct interaction with pathological proteins, enhanced autophagy, or other indirect effects.

To further clarify the actual contribution and translational value of natural product-derived CDs in regulating abnormal protein homeostasis, future studies should integrate quantitative assessments of brain distribution, measurements of pathological protein burden, validation of the autophagy–lysosomal pathway, long-term behavioral evaluation, and safety analysis.

### 4.3. Targeted Delivery and Controlled-Release Systems

In addition to their potential intrinsic neuroprotective effects, natural product-derived CDs (nanoparticles) and their functionalized, drug-loaded systems can serve as nanodelivery platforms, enhancing the transport of drugs or nucleic acids across the BBB and improving their delivery and release within the brain [212]. In NDDs, targeted delivery systems are designed to overcome the limited entry of therapeutic molecules into the CNS and insufficient exposure to the brain, whereas controlled-release systems can extend the duration of therapeutic action and reduce exposure to non-target tissues, thereby mitigating potential adverse effects to some extent [166].

Accordingly, the value of natural product-derived CDs in NDD delivery research lies not only in their potential to cross the BBB but also in their modifiable surface, drug-loading capacity, and suitability for responsive-release designs. It should be emphasized that BBB penetration, brain distribution, accumulation at pathological sites, and therapeutic benefit are distinct levels of evidence and should not be considered equivalent.

#### 4.3.1. Targeted Drug Delivery Systems

The advantages of CDs for brain delivery are primarily associated with their small size, good water solubility, and surface-modification capability. Their small size may facilitate tissue penetration and cellular uptake, while surface functional groups, such as hydroxyl, carboxyl, and amino groups, can improve colloidal stability and provide reactive sites for the modification of targeting ligands, drug conjugation, and the modulation of biological interfaces [24,213]. Unlike tumor delivery, where the enhanced permeability and retention effect is often emphasized, brain delivery in NDDs relies more heavily on interactions between materials and the BBB’s endothelial cells, transporters, and receptor systems [212,214,215]. Therefore, the brain-delivery capacity of CDs should be evaluated in terms of in vitro BBB permeability, fluorescence signals in brain tissue, brain parenchymal exposure, and accumulation in pathological regions.

Previous studies have provided some experimental evidence for the delivery of natural product-derived CDs across the BBB. For instance, fucoidan-derived CDs may retain functional groups, such as sulfate groups, and exhibit a negative surface charge. These CDs have been reported to cross the BBB and improve motor function in PD mice [216]. Radix Isatidis-derived CDs have also been shown to penetrate the BBB and produce therapeutic effects in an AD mouse model [33]. Additionally, glucose-derived fluorescent CDs have been reported to cross the BBB without additional targeting ligands, potentially via glucose transporter-mediated uptake [159]. Conversely, tryptophan-derived CDs can enter the CNS via the LAT1 transport system [161]. These findings suggest that surface groups derived from natural precursors or generated through carbonization-induced structural reconstruction may facilitate interactions between CDs and BBB transport systems.

To enable active targeting, the surfaces of CDs can be functionalized with hydroxyl, carboxyl, and amino groups to which glucose, amino acids, lactoferrin, or peptide ligands can be introduced, thereby enhancing their interactions with transport systems such as GLUT1, LAT1, and the lactoferrin receptor [159,215,217]. For instance, glucose- or trehalose-functionalized carbon-based polymers loaded with pDNA were able to cross the BBB via a GLUT1-related pathway to achieve nuclear gene delivery in a PD model [165]. Phenylalanine-functionalized CDs have also been reported to cross the BBB, inhibiting α-syn aggregation and alleviating neuroinflammation in the brains of PD mice [208]. Additionally, microglial membrane-biomimetic nanovesicles constructed from caffeic acid-conjugated CQDs could bypass BBB-related limitations via intranasal administration, thereby enhancing delivery to inflamed brain regions and offering a new design strategy for targeting AD-associated lesions [218].

Overall, natural product-derived CDs and their functionalized systems may improve brain-delivery potential through the possible effects of their small size on endothelial uptake and tissue diffusion, recognition mediated by surface groups derived from natural precursors, active targeting modifications, and biomimetic nose-to-brain delivery. These strategies provide a material basis for brain-targeted drug delivery in NDDs. However, most current studies still lack systematic analyses of quantitative brain distribution, brain-to-blood ratios, localization within pathological regions, and associations with long-term pharmacodynamic outcomes. Therefore, while the available findings support the potential of these systems for brain delivery, they should not be interpreted as evidence of stable accumulation at pathological sites or clinical therapeutic superiority.

#### 4.3.2. Stimuli-Responsive Controlled-Release Systems

In addition to targeted delivery, CDs derived from natural products and their functionalized systems can be used to load and control the release of drugs, nucleic acids, or naturally occurring bioactive molecules. Their small size, good water solubility, biocompatibility, and numerous surface functional groups (including hydroxyl, carboxyl, and amino groups) provide a basis for drug adsorption, covalent conjugation, nucleic acid complexation, and the construction of responsive structures [36,219,220]. In the treatment of NDDs, brain-targeted, controlled-release systems are relevant in two main ways. Firstly, strategies involving drug protection, carrier targeting, and trans-BBB transport may improve drug delivery and exposure within the brain. Secondly, elevated ROS levels, altered activities of inflammation-associated enzymes, or acidic endosomal and lysosomal environments in pathological regions can be exploited to achieve sustained or stimuli-responsive release. This prolongs local therapeutic activity and reduces off-target exposure [221,222,223,224].

Previous studies have reported CD-based systems for sustained drug release. For instance, chitosan-derived CDs can be combined with chitosan (CS) to form a CS/CD nanocarrier loaded with dopamine, creating a dopamine@CS/CD system. This system enables sustained dopamine release and uses the fluorescence properties of CDs to track delivery, indicating its potential for drug delivery in the nervous system [220]. Additionally, a curcumin delivery system based on an Fe_3_O_4_@carbon dot nanocomposite can enhance curcumin stability and cellular uptake, setting a precedent for the nanodelivery of naturally occurring bioactive compounds [225]. However, these studies mainly demonstrate the drug-loading and release capacities of CD-based systems. Whether such systems can effectively target the brain and demonstrate long-term therapeutic efficacy in NDD models remains to be validated.

For stimuli-responsive release, microenvironmental changes such as elevated ROS levels, inflammatory conditions, and acidic intracellular compartments may serve as potential triggers. A supramolecular carbon dot system constructed from lemon-derived CDs and a curcumin analog was able to load methotrexate and exhibit sustained release under acidic pH conditions. This provides a methodological reference for designing pH-responsive, controlled-release systems [226]. However, this study was not conducted in an NDD model and therefore cannot be extrapolated as therapeutic evidence for NDDs. In contrast, glucose/trehalose-functionalised carbon-based polymeric nanoparticles loaded with pDNA exhibited ROS-responsive behavior under oxidative stress, combining this property with GLUT1-mediated transport across the BBB to achieve nuclear gene delivery and functional cargo release in a PD model [165]. This study more directly demonstrates the potential of CD-related systems for responsive delivery in NDDs.

In summary, natural product-derived CDs and their functionalized systems may enable the stable loading and controlled release of drugs or nucleic acids through surface functional group-mediated interactions, covalent conjugation, non-covalent assembly, and pH- or ROS-responsive structural designs. These strategies could improve brain delivery efficiency and the release profiles of therapeutic agents, offering new approaches to long-term treatment and multimodal intervention in NDDs. However, the current evidence still has several notable limitations. First, some controlled-release systems have only been evaluated in non-NDD models and therefore mainly provide methodological guidance. Second, systematic evaluations of release kinetics in the brain, local drug concentrations at pathological sites, and long-term safety remain lacking. Third, the respective contributions of the carrier itself, the loaded therapeutic agent, and surface modifications have not been adequately distinguished.

Future studies should integrate quantitative pharmacokinetic analysis, regional brain distribution, release kinetics, long-term toxicological assessment, and correlations with therapeutic efficacy in disease models to further clarify the therapeutic value of controlled-release systems based on natural product-derived CDs for NDDs.

### 4.4. Theranostics and Visualization-Based Monitoring

In NDD research, theranostic materials can combine pathological recognition and delivery tracking with therapeutic intervention. This provides researchers with tools to investigate disease mechanisms and evaluate treatment efficacy. Some CD systems have exhibited low cytotoxicity in short-term in vitro studies, and have tunable fluorescence and surfaces that can be easily functionalized. These properties enable their use for imaging pathological structures, tracking carrier distribution, and delivering therapeutic agents. They can also be combined with anti-oxidative, anti-inflammatory, or anti-aggregation functions to create integrated systems that encompass ‘imaging and tracking–targeted delivery–therapeutic intervention’ [212,227,228].

However, it should be noted that fluorescence signals primarily reflect material distribution, binding to pathological structures or local response processes, and cannot be directly equated with effective brain exposure, therapeutic concentrations at pathological sites, or long-term therapeutic benefit [113,229]. Therefore, when evaluating the theranostic value of CDs, distinct levels of evidence, including imaging capability, brain delivery, pathological lesion recognition, and therapeutic efficacy, should be clearly differentiated.

#### 4.4.1. Fluorescence-Based Pathological Imaging

The imaging value of CDs derived from natural products primarily arises from their stable and tunable fluorescence properties. Their photoluminescence behavior is generally associated with the carbon core structure, surface states, molecular states, and fluorophore groups formed during precursor conversion. Their emission wavelengths can be tuned by adjusting the precursor composition, synthesis conditions, and surface functional groups. Red- and near-infrared (NIR)-emissive CDs can reduce interference from tissue autofluorescence and light scattering to some extent. This improves tissue penetration depth and imaging contrast, making them more suitable for deep-tissue or in vivo imaging [230,231,232,233,234,235,236]. For instance, protonated o-phenylenediamine-derived CDs demonstrate high-color-purity red emission and NIR two-photon emission, offering a valuable model for grasping fluorescence-regulation mechanisms in CDs [230]. However, this system is not derived from natural products and is therefore more appropriately regarded as a reference for mechanistic studies of CD fluorescence.

Among natural product-derived CDs, glutathione-derived NIR CDs exhibited low cytotoxicity in short-term in vitro assessments and possessed two-photon imaging capability, suggesting their potential for deep-tissue imaging [231]. Additionally, red- or NIR-emissive CDs derived from *Epigynum auritum*, spinach, lemon juice, mulberry leaves, and yew leaves have demonstrated favorable photostability, biocompatibility, and in vitro and in vivo imaging performance [232,233,234,235,236]. These studies suggest that red- and NIR-emissive, natural product-derived CDs could be used as fluorescent probes to track brain distribution, identify pathological structures, and design theranostic systems. However, most studies remain limited to general bioimaging or tissue imaging applications, and substantial evidence is lacking regarding the specific recognition of NDD-associated pathological lesions.

#### 4.4.2. Lesion Recognition and Dynamic Monitoring

CDs derived from natural products and their functionalized derivatives can interact with abnormal proteins or pathological microenvironments via their surface aromatic structures and functional groups, such as hydroxyl, carboxyl, and amino groups. This induces changes in fluorescence signals that can be used to recognize pathological structures and monitor their dynamics [209,237]. For instance, EGCG-derived carbonized polymer dots exhibit increased fluorescence upon binding to Aβ fibrils, thereby enabling Aβ fibril recognition [182]. Similarly, quercetin-derived red-emissive CDs exhibit a ‘turn-on’ red fluorescence upon contact with Aβ plaques, facilitating visualization of Aβ aggregates [204]. Similarly, resveratrol-derived CDs exhibit fluorescence responses to amyloid fibrils and can be used to monitor amyloid aggregation [238]. These findings suggest that CDs derived from naturally occurring bioactive molecules may enable recognition of pathological structures via binding-induced fluorescence changes to abnormal proteins.

Some natural product-derived CDs and their functionalized systems can also be used to track and visualize lesion-associated regions in vivo. Microglial membrane-biomimetic nanovesicles constructed from caffeic acid-conjugated CQDs can bypass BBB-related limitations upon intranasal administration and accumulate in inflamed brain regions. Their fluorescence signals enable the visualization of the in vivo distribution and delivery processes [218]. Fucoidan- and Radix Isatidis-derived CDs have both been reported to cross the BBB, exhibiting brain-delivery- and neuroprotection-related effects in PD and AD models [33,216]. In PD-related studies, phenylalanine-derived CDs have been reported to bind to α-syn monomers and oligomers and to penetrate the BBB, providing a basis for tracking and intervening in α-syn-associated pathological processes [208].

Overall, natural product-derived CDs and their functionalized systems may contribute to monitoring NDD-associated lesions through fluorescence responses, abnormal protein recognition, and brain-delivery tracking. However, most current studies have focused on Aβ fibrils, Aβ plaques, or model proteins. Furthermore, fluorescence signals may be influenced by material distribution, residual intravascular signal, tissue autofluorescence, and probe concentration. Therefore, fluorescence enhancement or signals detected in the brain alone cannot demonstrate localization within the brain parenchyma or the occurrence of a therapeutic effect.

#### 4.4.3. Integration of Theranostic Functions and Monitoring of Therapeutic Processes

In addition to lesion recognition, natural product-derived CDs and their functionalized systems can combine fluorescence tracking, brain-targeted delivery, and therapeutic intervention on a single platform. Their tunable fluorescence properties facilitate visualization of carrier distribution and delivery processes, while their surface modifiability and drug-loading capacity provide a structural basis for anti-aggregation, antioxidant, and anti-inflammatory functions, as well as drug delivery [24,37,38,239]. For instance, EGCG-derived carbonized polymer dots demonstrate enhanced fluorescence when they bind to Aβ fibrils, simultaneously inhibit Aβ fibrillization, promote the disassembly of mature fibrils, and scavenge ROS. This shows that they have a certain degree of integrated ‘recognition–intervention’ capability [182]. Quercetin-derived red-emissive CDs enable red-fluorescence imaging of Aβ plaques and exhibit anti-Aβ aggregation and antioxidant activities. This suggests their potential to recognize Aβ pathology and monitor therapeutic processes [204].

For delivery and tracking purposes, nanovesicles biomimetic of the microglial membrane, constructed from caffeic acid-conjugated CQDs, can be delivered to inflamed brain regions following intranasal administration. Their fluorescence signals enable visualization of in vivo distribution, brain-delivery processes, and accumulation within inflammatory regions [218]. Curcumin-loaded Fe_3_O_4_@CQD nanocomposites combine CQD-based fluorescence tracking with magnetically targeted delivery and improve curcumin cellular uptake, inhibiting Aβ aggregation and ROS-induced neurotoxicity [225]. Additionally, fucoidan- and phenylalanine-derived CDs have exhibited BBB-penetrating ability and improved motor function in PD models, providing experimental evidence for tracking brain delivery and evaluating efficacy [208,216].

In summary, CDs derived from natural products and their functionalized systems may contribute to the development of theranostic platforms for NDDs through fluorescence tracking, brain delivery, recognition of abnormal proteins, and anti-aggregation, antioxidant, and anti-inflammatory effects. These properties provide a material basis for lesion localization, visualization of carrier distribution, and monitoring of therapeutic processes. However, the available evidence should be interpreted cautiously. Fluorescence signals primarily reflect material distribution, binding to pathological structures, or local response processes, rather than directly demonstrating effective drug concentrations in the brain, selective accumulation within pathological lesions, or long-term disease-modifying effects.

Table 3 summarizes the relevant preclinical studies by preparation methods, disease models, evidence of BBB penetration or brain delivery, major outcomes, and study limitations.

Taken together, the studies listed in Table 3 suggest that the brain exposure, in vivo stability, metabolism and clearance, tissue accumulation, and long-term safety of natural product-derived CDs have not yet been systematically evaluated. Given the chronic progression of NDDs and the need for long-term treatment, future studies should extend beyond functional validation and strengthen systematic assessments of pharmacokinetics, biodistribution, safety, quality control, and translational feasibility. The following section discusses the major challenges facing natural product-derived CDs with respect to absorption, distribution, metabolism, and excretion (ADME), long-term safety, material consistency, and clinical translation within these evidentiary boundaries.

## 5. Translational Challenges and Preclinical Evaluation of Natural Product-Derived Carbon Dots

The translational potential of CDs derived from natural products cannot be assessed solely on the basis of short-term functions such as antioxidant and anti-inflammatory effects, inhibition of protein aggregation, or imaging and tracking capabilities. Instead, they should be evaluated comprehensively in conjunction with their in vivo disposition, exposure to the brain parenchyma, long-term safety, material consistency, and validation in human-relevant models [92,98,127]. As current studies largely rely on simplified BBB models and short-term animal experiments, in vitro permeability, signals detected in brain tissue, and functional improvements alone are insufficient to demonstrate effective exposure within the brain parenchyma [112,113,240,241].

### 5.1. ADME Characteristics and Evaluation of Brain Exposure

The circulation kinetics, biodistribution, and clearance of CDs can be markedly influenced by different routes of administration. In addition, the protein corona formed upon entry into the bloodstream can alter their surface properties, circulation time, immune recognition, and in vivo fate [119,242]. While oral administration is convenient for long-term treatment, its efficiency for brain delivery is limited by gastrointestinal stability, intestinal absorption, first-pass metabolism, and systemic bioavailability [243]. Following intranasal administration, CDs can access the CNS via the olfactory, trigeminal, and perivascular pathways. However, distribution to the brain after intranasal delivery should not be considered equivalent to conventional BBB penetration, and direct nose-to-brain transport should be distinguished from brain distribution following systemic absorption [244,245,246].

Assessment of brain exposure should not rely solely on fluorescence signals from the whole brain or brain sections. These signals may be affected by residual intravascular material, inadequate perfusion, binding to or uptake by brain microvascular endothelial cells, leakage, or degradation of fluorescent labels. Therefore, they cannot independently demonstrate entry into the brain parenchyma or attainment of an effective concentration at pathological sites [113,229,247]. Brain tissue-associated total signal, brain parenchymal exposure, and exposure at the site of action must therefore be distinguished and evaluated comprehensively using time-course measurements of brain-to-blood ratios, adequate perfusion or vascular correction, capillary depletion, regional and cellular localization, and quantitative tissue analysis [112,113,248]. For CDs containing stable characteristic elements or radiolabels, in vivo tracking and quantification may be feasible; however, the label’s in vivo behavior must remain consistent with that of the CD itself. Methods such as liquid chromatography can be used to analyze free fluorophores, residual precursors, and low-molecular-weight by-products. However, in the absence of particle-specific separation and identification, the detected signals should not be directly interpreted as quantitative measurements of the CD itself [249,250,251].

The in vivo distribution, transformation, and clearance pathways of CDs remain to be fully clarified. Their clearance behavior cannot be inferred solely from dry-state particle size measured by transmission electron microscopy (TEM) but should also be evaluated in relation to hydrodynamic size, aggregation state, surface charge, and surface modification. Well-dispersed CDs with small hydrodynamic diameters may be partially cleared via renal excretion, whereas cross-linked or aggregated multi-CD nanoparticles may be predominantly cleared via the hepatobiliary system [242,252,253,254]. In addition to tissue distribution and excretion, it is essential to determine whether fluorescent labels can stably represent the CD itself. Dye leakage, fluorescence quenching or dequenching, signal saturation, and labeling-induced changes in cellular uptake may all confound the interpretation of in vivo distribution [92,113,229]. For drug- or nucleic acid-loaded systems, the in vivo behavior of the carrier, label, and therapeutic payload should be evaluated separately to avoid inferring drug release or effective brain exposure solely from carrier fluorescence. Future studies should incorporate time-dependent measurements in blood, urine, feces, and major organs to systematically characterize the in vivo fate of CDs and their associated components.

### 5.2. Long-Term Safety, Immunocompatibility, and Neurotoxicology

The treatment of NDDs generally requires long-term or repeated administration; therefore, favorable short-term tolerability or low short-term cytotoxicity does not guarantee long-term safety. For nanomaterials with the potential to be delivered to the brain, safety assessments should address not only toxicity in peripheral organs but also the risks of oxidative stress, deoxyribonucleic acid (DNA) damage, neuroinflammation, and intracerebral accumulation following BBB translocation [98,255,256]. Previous studies have shown that some CDs exhibit low toxicity in vitro yet cause significant in vivo toxicity following repeated administration [175]. CD-based nanoparticles characterized by prolonged retention or hepatobiliary clearance also require further evaluation of their long-term nanotoxicological risks [254].

It should be emphasized that the historical medicinal use or natural origin of a precursor cannot be regarded as evidence that the resulting CDs are non-toxic or immunocompatible. Carbonization can substantially alter the original constituents, generating new carbon cores, surface structures, and low-molecular-weight by-products. Therefore, safety studies should evaluate the final CD product after adequate purification and characterization, rather than relying on the medicinal history or natural origin of the precursor as a substitute for systematic toxicological evidence [28,230,257,258].

Future studies should systematically assess hematological parameters, coagulation function, hemolysis, complement activation, inflammatory mediators, oxidative stress, liver and kidney function, and histopathological alterations in major organs under conditions of repeated administration and long-term exposure [259,260,261]. For brain-delivery systems in particular, attention should be paid to nanocarrier-induced neuroinflammation, alterations in BBB homeostasis, intracerebral accumulation, and chronic neurotoxicity. Relevant endpoints should include neuronal injury, synaptic structure and function, glial activation, and cerebrovascular integrity [98]. Only by integrating the evaluation of peripheral toxicity, CNS safety, and long-term immunocompatibility can the suitability of CDs derived from natural products for treating chronic NDDs be assessed more accurately.

### 5.3. Material Reproducibility, Validation Using Human-Relevant Models, and Regulatory Translation

Material reproducibility is a critical prerequisite for the translational application of CDs derived from natural products. Natural product precursors are compositionally complex, and the source of the precursors, the conditions under which they are prepared, and the purification procedures can all affect the composition, surface properties, fluorescence behavior, and biological activity of the resulting CDs [28,258,262,263]. Inadequate purification can also result in the presence of low-molecular-weight components, nanoscale aggregates, or fluorescent impurities, leading to an unclear material identity and incorrect attribution of biological effects [257,258]. Candidate systems with translational potential should therefore establish a systematic quality-control framework that standardizes precursor sources, preparation parameters, purification procedures, and batch-to-batch consistency. Key quality attributes, including particle size and size distribution, surface charge and chemical composition, optical properties, purity and stability in biological media, should be adequately characterized [264,265,266]. Biological experiments should also include controls consisting of the precursor, low-molecular-weight fractions, and adequately purified CDs in order to distinguish the relative contributions of the CD material itself, residual precursors, and by-products.

Differences in gene expression, transporter profiles, and receptor-mediated transcytosis systems between the human and rodent BBB may limit the clinical predictive value of animal brain-delivery findings [267,268,269]. In addition, BBB/NVU dysfunction associated with aging and NDDs may alter the brain distribution, clearance, and safety of these materials [270,271]. Therefore, future studies should incorporate platforms such as human-induced pluripotent stem cell (iPSC)-derived BBB/NVU models, microfluidic barrier-on-chip systems, and vascularized brain organoids to evaluate the brain-delivery capacity and central nervous system safety of natural product-derived CDs [272,273,274].

Based on the available preclinical evidence, CDs derived from Radix Isatidis, fucoidan, phenylalanine, and other naturally occurring bioactive molecules have been evaluated in disease models, demonstrating therapeutic efficacy and BBB-associated delivery. These CDs therefore currently have comparatively greater preclinical support. However, existing studies generally lack quantification of brain parenchymal exposure specific to individual particles, systematic pharmacokinetic characterization, repeated-dose safety assessment, evaluation of batch-to-batch consistency, and validation in human-relevant BBB models [33,182,208,216]. Consequently, no natural product-derived CD system can yet be identified as having a clear translational advantage, and the field remains largely at the proof-of-concept stage.

From a regulatory perspective, the development category of a candidate product should be defined according to its composition and intended use. This includes whether it is being developed as an active nanomaterial, a drug delivery system, an imaging probe, or a theranostic combination product. Preclinical development should also clarify the identity of the materials and the basis of the active components, establish key quality attributes and exposure–response relationships, and generate evidence to support dose selection, Good Laboratory Practice (GLP)-compliant toxicology, Good Manufacturing Practice (GMP)-compliant scale-up production, and product batch consistency [275,276,277,278,279].

**Figure 4:**
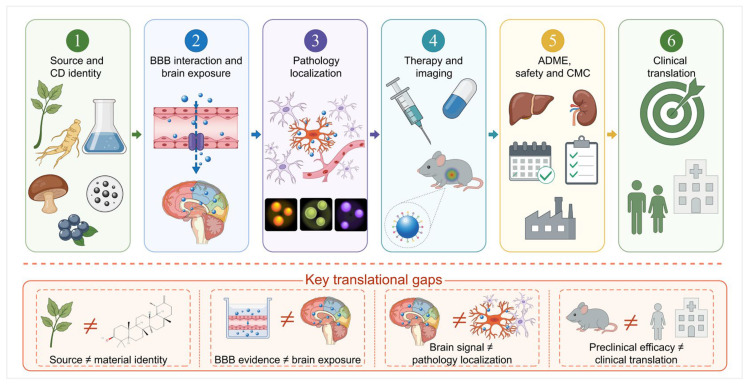
Evidence chain and key translational gaps for natural product-derived carbon dots from material identity to clinical translation. Translational evaluation encompasses material identity, BBB-associated delivery and brain parenchymal exposure, localization within pathological regions, therapeutic efficacy and imaging performance, safety and CMC requirements, and regulatory and clinical translation considerations. Arrows indicate the sequential progression of the translational evidence chain, whereas colors distinguish the six evaluation stages and do not indicate quantitative differences. Evidence obtained at these different levels should not be considered interchangeable. Abbreviations: ADME, absorption, distribution, metabolism, and excretion; BBB, blood–brain barrier; CDs, carbon dots; CMC, chemistry, manufacturing, and controls.

Overall, the translational evaluation of natural product-derived CDs requires a continuous chain of evidence extending from material quality and BBB-associated delivery to brain parenchymal exposure, pathological localization, attribution of pharmacological effects, long-term safety, validation in human-relevant models, and standardized manufacturing. As illustrated in Figure 4, each stage should be validated sequentially. A natural precursor, in vitro BBB permeability, a signal detected in brain tissue, or therapeutic efficacy in an animal model should not be considered equivalent to a compositionally defined material, effective brain parenchymal exposure, lesion-specific targeting, or clinical translational value, respectively.

## 6. Conclusions 

Overall, CDs derived from natural products represent a class of candidate materials with exploratory value for brain delivery, neuroprotection, and theranostic research in NDDs. Preclinical studies indicate that their small size, tunable surface chemistry, and functional modifications could facilitate interaction with the BBB. Some systems have also been shown to exhibit antioxidant, anti-inflammatory, and abnormal protein aggregation-modulating activities, as well as drug delivery and fluorescence tracking in cellular and animal models. Therefore, natural product-derived CDs may support the development of multifunctional nanotheranostic strategies that integrate ‘delivery–intervention–monitoring’ for NDDs. However, it should be emphasized that in vitro BBB permeability, fluorescence signals in brain tissue, brain-associated distribution, brain parenchymal exposure, and therapeutic benefit are distinct levels of evidence and should not be considered interchangeable.

The further translation of natural product-derived CDs is constrained by multiple factors. Firstly, their material composition, structural characteristics, and consistency across batches require further clarification. Because natural product precursors are compositionally complex, carbonization conditions, purification procedures, surface states, and residual small molecules may all influence the structural characteristics and biological effects of the resulting CDs. Reproducible manufacturing processes must therefore be established, and key quality attributes and impurity-control specifications must be defined. Secondly, there is insufficient evidence regarding brain delivery and ADME. Brain fluorescence signals or behavioral improvements alone cannot demonstrate effective exposure of the brain parenchyma. Future studies should therefore incorporate quantitative brain distribution measurements, brain-to-blood ratios, capillary depletion analysis, regional brain localization, pharmacokinetics, metabolic transformation, and hepatic and renal clearance to clarify the relationships among route of administration, brain exposure, and therapeutic efficacy. Thirdly, long-term efficacy and safety require systematic validation, paying particular attention to tissue accumulation, immune responses, neurotoxicity, hematocompatibility, clearance kinetics and chronic nanotoxicological risks following repeated administration.

Future research should move beyond functional proof of concept toward a systematic evaluation oriented toward clinical translation. Structurally defined, adequately purified, and batch-consistent CD systems should be used to clarify the influence of particle size, surface charge, functional groups, heteroatom doping, and residual precursor components on BBB interactions and neuroprotective effects. In addition, more clinically relevant, human-derived BBB models, NVU models, organ-on-chip platforms, and multicellular co-culture systems should be introduced to assess delivery efficiency, barrier safety, and immunological effects in human-relevant barrier environments. Meanwhile, representative NDD models should be used to establish integrated evaluation frameworks encompassing brain exposure, pathological endpoints, behavioral outcomes, and long-term safety. Natural product-derived CDs can only progress beyond the proof-of-concept stage toward nanomedicine platforms with genuine translational potential for neurological applications when material composition is controllable, brain parenchymal exposure is clearly demonstrated, exposure–efficacy relationships are established, and long-term safety is acceptable.

## Figures and Tables

**Table 1:** Molecular Pathology, Affected Brain Regions, and Clinical Features of Major Neurodegenerative Diseases.

Disease	Representative Abnormal Protein/Molecule	Main Affected Brain Region/Cells	Typical Pathological Features	Major Clinical Manifestations
Alzheimer’s Disease (AD) [1,6,67]	Aβ, Tau	Hippocampus, entorhinal cortex, cerebral cortex	Extracellular Aβ plaque deposition; Tau hyperphosphorylation forming neurofibrillary tangles; synaptic damage and neuronal loss	Memory decline, cognitive impairment, executive dysfunction
Parkinson’s Disease (PD) [53,68,69]	α-syn	Substantia nigra pars compacta, striatum, dopaminergic neurons	α-syn abnormal aggregation forming Lewy bodies; degeneration of nigral dopaminergic neurons	Resting tremor, bradykinesia, rigidity, postural instability
Huntington’s Disease (HD) [3,70,71]	mHTT	Striatum, cortex	CAG repeat expansion in *HTT* gene; mHTT aggregation; selective loss of medium spiny neurons in striatum	Choreiform movements, psychiatric and behavioral disturbances, cognitive decline
Amyotrophic Lateral Sclerosis (ALS) [4,72,73]	TDP-43, SOD1, FUS, *C9orf72*-related abnormalities	Upper and lower motor neurons; some glial cells	Cytoplasmic mislocalization and inclusions of TDP-43; RNA metabolism dysregulation; axonal transport defects	Progressive muscle weakness, atrophy, swallowing and respiratory dysfunction
Frontotemporal Dementia (FTD) [74,75]	Tau, TDP-43, FUS	Frontal and temporal lobes	Frontal and temporal lobe atrophy; subtype-specific protein inclusions	Behavioral changes, personality alterations, language impairment
Dementia with Lewy Bodies (DLB) [76,77,78]	α-syn, often with Aβ/Tau	Cerebral cortex, limbic system, brainstem	Widespread Lewy body deposition; mixed pathologies common	Cognitive fluctuations, visual hallucinations, parkinsonism
Multiple System Atrophy (MSA) [79,80,81]	α-syn	Oligodendrocytes, basal ganglia, cerebellum, brainstem	Glial cytoplasmic inclusions; widespread involvement of autonomic and motor systems	Cerebellar ataxia, parkinsonian syndrome, autonomic dysfunction
Progressive Supranuclear Palsy (PSP) [82,83,84]	Tau	Brainstem, basal ganglia, frontal lobe	4R-Tau proteinopathy; Tau accumulation in neurons and glia	Vertical gaze palsy, postural instability, bradykinesia
Corticobasal Degeneration (CBD) [85,86]	Tau	Fronto-parietal cortex, basal ganglia	Asymmetric Tau deposition in cortex and basal ganglia; degeneration of cortico-basal ganglia circuits	Limb apraxia, dystonia, cognitive and motor impairments

**Table 2:** Classification, Representative Precursors, Structural Characteristics, Reported Functional Properties, and Key Biomedical Features of Natural Product-Derived Carbon Dots.

Category	Representative Precursors/Sources	Key Structural Characteristics	Reported Functional Properties	Key Biomedical Features and Implications
Carbon dots derived from natural small molecules [132,133]	Flavonoids [134]; diterpenoid lactones [135]	sp^2^ carbon core retaining partial π-conjugated structures and molecular skeleton features; surface enriched with –OH/–COOH groups	Antioxidant activity, fluorescence imaging, metal ion detection	Small-molecule precursors provide high structural tunability, enabling precise regulation of optical and antioxidant properties via molecular design and carbonization degree
Carbon dots derived from biomacromolecules [136]	Polysaccharides [137,138,139]; proteins [140,141]	Three-dimensional crosslinked carbon network enriched with high-density –OH/–COOH/–NH_2_ functional groups	High biocompatibility; antioxidant, antibacterial, imaging, and drug delivery capabilities	Macromolecular precursors offer abundant functional groups and excellent biocompatibility, conferring significant advantages in biomedical and theranostic applications
Food-derived carbon dots [142]	Honey [143]; tomato [144]	sp^2^ carbon core often with heteroatom (e.g., N) doping and abundant surface states	Fluorescence emission, antioxidant, antibacterial, bioimaging	Potential biocompatibility and sustainability; heteroatom doping may enhance optical properties and bioactivity
Carbon dots derived from medicinal plant extracts [145,146]	*Salvia miltiorrhiza* [147]; *Lycium barbarum* [148]; Aconite charcoal-derived CDs [149]	sp^2^ carbon core retaining bioactive molecular fragments or polymerized structures; surface rich in oxygen-containing groups	Antioxidant, anti-inflammatory, antibacterial, with potential antitumor and metabolic regulatory effects	Retention of precursor-related features and potential integration of nanomaterial and biological functions
Carbon dots from carbonized natural products [150]	Carbonized *Cirsium* [151]; carbonized madder (*Rubia cordifolia*) [152]	Highly graphitized carbon core enriched with C=O and –OH groups, forming specific active surface sites	Hemostatic and procoagulant effects (activation of extrinsic coagulation pathway, increased fibrinogen, enhanced platelet function)	Carbonization generates key active components such as CDs, which regulate coagulation pathways and fibrinogen systems, forming the material basis of hemostatic effects
Composite natural-source carbon dots	Algal polysaccharides with betaine [153]; red ginseng root extract with rutin [154]; pollen, lotus node, *Cirsium*, and human hair [155]; curcumin with citric acid [156]	Multi-precursor co-carbonization forming carbon cores enriched with –OH/–COOH/–NH_2_ groups; characterized by multi-element synergistic doping and complex surface structures	Antioxidant, antibacterial, anti-inflammatory, hemostatic, and tissue repair-promoting activities	Synergistic carbonization of multiple precursors integrates structure and function, significantly enhancing antibacterial, antioxidant, and tissue repair performance via multi-component cooperative mechanisms

**Table 3:** Representative studies of natural product-derived carbon dots in neurodegenerative disease-related models.

Precursor/Source	Synthesis Method	Disease/Model	BBB/Brain-Delivery Evidence	Main Outcomes	Key Limitations
Radix Isatidis-derived carbon dots [33]	Hydrothermal synthesis	Aβ42- and LPS-induced cellular models; LPS-induced AD-like mouse model	Cy5.5 brain fluorescence suggested possible BBB penetration	Reduced ROS, Aβ42 aggregation, and inflammatory responses, and improved cognitive performance	Brain fluorescence does not demonstrate brain parenchymal exposure; the model mainly reflects inflammation-driven AD-like phenotypes
Fucoidan-derived carbon dots [216]	One-step hydrothermal synthesis	MPP^+^/H_2_O_2_-induced PC12 cell injury; MPTP-induced PD-like mouse model	Cy5.5 brain fluorescence suggested possible BBB penetration	Reduced oxidative stress, apoptosis, and mitochondrial injury, and improved motor performance	BBB evidence relied mainly on fluorescence imaging; the animal study used preventive and concurrent intervention
Glucose-derived carbon dots [159,160]	Hydrothermal carbonization of glucose; fluorescein conjugation for tracing	Yeast transporter model; zebrafish and rat CNS delivery models	GluCD-F signals were detected in the CNS; uptake may involve glucose transporters	Supported CNS delivery, neuronal imaging, and neural network tracing	Evidence was mainly limited to delivery and imaging; direct NDDs therapeutic outcomes and quantitative brain exposure data were lacking
Phenylalanine/urea-derived carbon dots [208]	Hydrothermal synthesis	α-Syn/PFF-related cellular models; MPTP-induced PD-like mouse model	An in vitro Transwell model suggested BBB permeability	Inhibited α-syn aggregation, reduced oxidative stress and inflammation, and improved motor performance	BBB evidence was limited to an in vitro model; the animal study used a preventive intervention design
EGCG/o-phenylenediamine-derived carbonized polymer dots [182]	Hydrothermal synthesis	Aβ aggregation and cytotoxicity models; AD nematode model	No clear BBB or brain-delivery evidence was reported	Inhibited and disassembled Aβ fibrils, scavenged ROS, reduced cytotoxicity, and prolonged nematode lifespan	Evidence was mainly limited to in vitro and nematode models; mammalian validation was lacking
Curcumin-derived carbon quantum dots [34]	One-step dry-heating synthesis	Aβ42 aggregation model; OA-induced tau hyperphosphorylation cell model	No clear BBB or brain-delivery evidence was reported	Inhibited Aβ42 aggregation and tau hyperphosphorylation, while improving aqueous solubility and cytocompatibility	Evidence was limited to in vitro studies; animal efficacy and brain delivery remain unverified

Note: Brain fluorescence, in vitro BBB permeability, brain tissue distribution, brain parenchymal exposure, and therapeutic benefit represent distinct levels of evidence and should not be considered interchangeable. Most studies still lack systematic evaluation of brain exposure, pharmacokinetics, clearance, and long-term safety.

## Data Availability

No new datasets were generated in this study. All data and information discussed in this review are available from the cited publications.

## Abbreviations

The following abbreviations are used in this manuscript:

α-syn	Alpha-synuclein
TS-IM	Fluorescence imaging-guided theranostics
TNF-α	Tumor necrosis factor-α
TLR4	Toll-like receptor 4
TJ	Tight junction
TEM	Transmission electron microscopy
TDP-43	TAR DNA-binding protein 43
TDDS-CDs	Carbon dot-based targeted drug delivery system
SOD	Superoxide dismutase
*SNCA*	Synuclein alpha gene
ROS	Reactive oxygen species
RMT	Receptor-mediated transcytosis
pDNA	Plasmid DNA
PD	Parkinson’s disease
NVU	Neurovascular unit
NO	Nitric oxide
NLRP3	NOD-like receptor family pyrin domain-containing 3
NIR	Near-infrared
NF-κB	Nuclear factor kappa B
NDDs	Neurodegenerative diseases
LPS	Lipopolysaccharide
LAT1	L-type amino acid transporter 1
IL-6	Interleukin-6
IL-1β	Interleukin-1β
IL-18	Interleukin-18
IAPA-PPD	Inhibition of abnormal protein aggregation
GSDMD	Gasdermin D
GQDs	Graphene quantum dots
GLUT1	Glucose transporter 1
FUS	Fused in sarcoma
EGCG	Epigallocatechin gallate
DNA	Deoxyribonucleic acid
CS	Chitosan
CQDs	Carbon quantum dots
CNS	Central nervous system
CMC	Chemistry, manufacturing, and controls
CDs	Carbon dots
BBB	Blood–brain barrier
Aβ	Amyloid-β
AOS-AIR	Antioxidative and anti-inflammatory regulation
AMT	Adsorptive-mediated transcytosis
ALS	Amyotrophic lateral sclerosis
ADME	Absorption, distribution, metabolism, and excretion
AD	Alzheimer’s disease
ABC	ATP-binding cassette

## References

[1] Scheltens P., De Strooper B., Kivipelto M., Holstege H., Chételat G., Teunissen C.E., Cummings J., van der Flier W.M. (2021). Alzheimer’s disease. Lancet.

[2] Tolosa E., Garrido A., Scholz S.W., Poewe W. (2021). Challenges in the diagnosis of Parkinson’s disease. Lancet Neurol..

[3] Jiang A., Handley R.R., Lehnert K., Snell R.G. (2023). From Pathogenesis to Therapeutics: A Review of 150 Years of Huntington’s Disease Research. Int. J. Mol. Sci..

[4] Feldman E.L., Goutman S.A., Petri S., Mazzini L., Savelieff M.G., Shaw P.J., Sobue G. (2022). Amyotrophic lateral sclerosis. Lancet.

[5] Cummings J. (2017). Disease modification and Neuroprotection in neurodegenerative disorders. Transl. Neurodegener..

[6] Jucker M., Walker L.C. (2023). Alzheimer’s disease: From immunotherapy to immunoprevention. Cell.

[7] Breijyeh Z., Karaman R. (2020). Comprehensive Review on Alzheimer’s Disease: Causes and Treatment. Molecules.

[8] Zheng Q., Wang X. (2025). Alzheimer’s disease: Insights into pathology, molecular mechanisms, and therapy. Protein Cell.

[9] Kulcsarova K., Skorvanek M., Postuma R.B., Berg D. (2024). Defining Parkinson’s Disease: Past and Future. J. Park. Dis..

[10] Tong H., Yang T., Xu S., Li X., Liu L., Zhou G., Yang S., Yin S., Li X.-J., Li S. (2024). Huntington’s Disease: Complex Pathogenesis and Therapeutic Strategies. Int. J. Mol. Sci..

[11] Rizea R.E., Corlatescu A.-D., Costin H.P., Dumitru A., Ciurea A.V. (2024). Understanding Amyotrophic Lateral Sclerosis: Pathophysiology, Diagnosis, and Therapeutic Advances. Int. J. Mol. Sci..

[12] Jia L., Quan M., Fu Y., Zhao T., Li Y., Wei C., Tang Y., Qin Q., Wang F., Qiao Y. (2020). Dementia in China: Epidemiology, clinical management, and research advances. Lancet Neurol..

[13] Passeri E., Elkhoury K., Morsink M., Broersen K., Linder M., Tamayol A., Malaplate C., Yen F.T., Arab-Tehrany E. (2022). Alzheimer’s Disease: Treatment Strategies and Their Limitations. Int. J. Mol. Sci..

[14] Wolfson C., Gauvin D.E., Ishola F., Oskoui M. (2023). Global Prevalence and Incidence of Amyotrophic Lateral Sclerosis: A Systematic Review. Neurology.

[15] Jiang Q., Liu J., Huang S., Wang X.-Y., Chen X., Liu G.-H., Ye K., Song W., Masters C.L., Wang J. (2025). Antiageing strategy for neurodegenerative diseases: From mechanisms to clinical advances. Signal Transduct. Target. Ther..

[16] Yu X., Ji C., Shao A. (2020). Neurovascular Unit Dysfunction and Neurodegenerative Disorders. Front. Neurosci..

[17] Argueti-Ostrovsky S., Alfahel L., Kahn J., Israelson A. (2021). All Roads Lead to Rome: Different Molecular Players Converge to Common Toxic Pathways in Neurodegeneration. Cells.

[18] Agnello L., Ciaccio M. (2022). Neurodegenerative Diseases: From Molecular Basis to Therapy. Int. J. Mol. Sci..

[19] Akhtar A., Andleeb A., Waris T.S., Bazzar M., Moradi A.-R., Awan N.R., Yar M. (2021). Neurodegenerative diseases and effective drug delivery: A review of challenges and novel therapeutics. J. Control. Release.

[20] Saraiva C., Praça C., Ferreira R., Santos T., Ferreira L., Bernardino L. (2016). Nanoparticle-mediated brain drug delivery: Overcoming blood-brain barrier to treat neurodegenerative diseases. J. Control. Release.

[21] Wang Z., Gonzalez K.M., Cordova L.E., Lu J. (2023). Nanotechnology-empowered therapeutics targeting neurodegenerative diseases. Wiley Interdiscip. Rev. Nanomed. Nanobiotechnol..

[22] Cano A., Muñoz-Morales Á., Sánchez-López E., Ettcheto M., Souto E.B., Camins A., Boada M., Ruíz A. (2023). Exosomes-Based Nanomedicine for Neurodegenerative Diseases: Current Insights and Future Challenges. Pharmaceutics.

[23] Liu J., Li R., Yang B. (2020). Carbon Dots: A New Type of Carbon-Based Nanomaterial with Wide Applications. ACS Cent. Sci..

[24] Zhang W., Sigdel G., Mintz K.J., Seven E.S., Zhou Y., Wang C., Leblanc R.M. (2021). Carbon Dots: A Future Blood-Brain Barrier Penetrating Nanomedicine and Drug Nanocarrier. Int. J. Nanomed..

[25] Zhao Y., Li Y., Li D., Yuan H., Shen C. (2025). Eco-Friendly Synthesized Carbon Dots from Chinese Herbal Medicine: A Review. Int. J. Nanomed..

[26] Kucharz K., Kristensen K., Johnsen K.B., Lund M.A., Lønstrup M., Moos T., Andresen T.L., Lauritzen M.J. (2021). Post-capillary venules are the key locus for transcytosis-mediated brain delivery of therapeutic nanoparticles. Nat. Commun..

[27] Xie J., Shen Z., Anraku Y., Kataoka K., Chen X. (2019). Nanomaterial-based blood-brain-barrier (BBB) crossing strategies. Biomaterials.

[28] Luo W.-K., Zhang L.-L., Yang Z.-Y., Guo X.-H., Wu Y., Zhang W., Luo J.-K., Tang T., Wang Y. (2021). Herbal medicine derived carbon dots: Synthesis and applications in therapeutics, bioimaging and sensing. J. Nanobiotechnol..

[29] Sahana S., Gautam A., Singh R., Chandel S. (2023). A recent update on development, synthesis methods, properties and application of natural products derived carbon dots. Nat. Prod. Bioprospect..

[30] Rigodanza F., Burian M., Arcudi F., Đorđević L., Amenitsch H., Prato M. (2021). Snapshots into carbon dots formation through a combined spectroscopic approach. Nat. Commun..

[31] Yao X., Lewis R.E., Haynes C.L. (2022). Synthesis Processes, Photoluminescence Mechanism, and the Toxicity of Amorphous or Polymeric Carbon Dots. Acc. Chem. Res..

[32] Bian Z., Wallum A., Mehmood A., Gomez E., Wang Z., Pandit S., Nie S., Link S., Levine B.G., Gruebele M. (2023). Properties of Carbon Dots versus Small Molecules from “Bottom-up” Synthesis. ACS Nano.

[33] Liu Z., Wang J., Chen Z., Shi C., Tang Z., Wang Y. (2026). BBB-permeable carbon dots ameliorate Alzheimer’s-like phenotypes in mice by suppressing oxidative stress, neuroinflammation, and amyloid-β aggregation. Int. Immunopharmacol..

[34] Lim J.L., Lin C.-J., Huang C.-C., Chang L.-C. (2024). Curcumin-derived carbon quantum dots: Dual actions in mitigating tau hyperphosphorylation and amyloid beta aggregation. Colloids Surf. B.

[35] Zeng M., Wang Y., Liu M., Wei Y., Wen J., Zhang Y., Chen T., He N., Fan P., Dai X. (2023). Potential Efficacy of Herbal Medicine-Derived Carbon Dots in the Treatment of Diseases: From Mechanism to Clinic. Int. J. Nanomed..

[36] Singh H., Razzaghi M., Ghorbanpoor H., Ebrahimi A., Avci H., Akbari M., Hassan S. (2025). Carbon dots in drug delivery and therapeutic applications. Adv. Drug Deliv. Rev..

[37] Iannazzo D., Celesti C., Cardo L., Prato M., Bitto A. (2026). Carbon dots for drug delivery: Insights into their potential in nanopharmacology. Pharmacol. Rev..

[38] Araújo C., Rodrigues R.O., Bañobre-López M., Silva A.M.T., Ribeiro R.S. (2025). Carbon Dots as a Fluorescent Nanosystem for Crossing the Blood-Brain Barrier with Plausible Application in Neurological Diseases. Pharmaceutics.

[39] Ullal N., Mehta R., Sunil D. (2024). Separation and purification of fluorescent carbon dots—An unmet challenge. Analyst.

[40] Sweeney M.D., Sagare A.P., Zlokovic B.V. (2018). Blood-brain barrier breakdown in Alzheimer disease and other neurodegenerative disorders. Nat. Rev. Neurol..

[41] Gambino C.M., Sasso B.L., Bivona G., Agnello L., Ciaccio M. (2019). Aging and Neuroinflammatory Disorders: New Biomarkers and Therapeutic Targets. Curr. Pharm. Des..

[42] Cullinane P.W., Wrigley S., Bezerra Parmera J., Valerio F., Millner T.O., Shaw K., De Pablo-Fernandez E., Warner T.T., Jaunmuktane Z. (2024). Pathology of neurodegenerative disease for the general neurologist. Pract. Neurol..

[43] Forrest S.L., Kovacs G.G. (2025). Protein Pathologies in Oligodendroglia in Neurodegenerative Diseases. Adv. Neurobiol..

[44] Therriault J., Zimmer E.R., Benedet A.L., Pascoal T.A., Gauthier S., Rosa-Neto P. (2022). Staging of Alzheimer’s disease: Past, present, and future perspectives. Trends Mol. Med..

[45] Wilson D.M., Cookson M.R., Van Den Bosch L., Zetterberg H., Holtzman D.M., Dewachter I. (2023). Hallmarks of neurodegenerative diseases. Cell.

[46] Long J.M., Holtzman D.M. (2019). Alzheimer Disease: An Update on Pathobiology and Treatment Strategies. Cell.

[47] Nichols J.B., Malek-Ahmadi M., Tariot P.N., Serrano G.E., Sue L.I., Beach T.G. (2021). Vascular Lesions, APOE ε4, and Tau Pathology in Alzheimer Disease. J. Neuropathol. Exp. Neurol..

[48] Forrest S.L., Kovacs G.G. (2023). Current Concepts of Mixed Pathologies in Neurodegenerative Diseases. Can. J. Neurol. Sci..

[49] Jang E.-H., Choi H., Hur E.-M. (2024). Microtubule function and dysfunction in the nervous system. Mol. Cells.

[50] Mathieu C., Pappu R.V., Taylor J.P. (2020). Beyond aggregation: Pathological phase transitions in neurodegenerative disease. Science.

[51] Chong Z.Z., Souayah N. (2025). Oxidative Stress: Pathological Driver in Chronic Neurodegenerative Diseases. Antioxidants.

[52] Kato Y., Kanatani M., Adachi T., Nagamine H., Horikoshi Y., Nakaso K. (2025). Oxidative modification of methionine residue in α-synuclein by dopamine induces cellular vulnerability, oligomerization and secretion of α-synuclein. J. Clin. Biochem. Nutr..

[53] Park H., Kam T.-I., Dawson V.L., Dawson T.M. (2025). α-synuclein pathology as a target in neurodegenerative diseases. Nat. Rev. Neurol..

[54] Jellinger K.A. (2025). Concomitant Pathologies and Their Impact on Parkinson Disease: A Narrative Overview of Current Evidence. Int. J. Mol. Sci..

[55] Hu Y., Chen W., Wei C., Jiang S., Li S., Wang X., Xu R. (2024). Pathological mechanisms of amyotrophic lateral sclerosis. Neural Regen. Res..

[56] Rummens J., Da Cruz S. (2025). RNA-binding proteins in ALS and FTD: From pathogenic mechanisms to therapeutic insights. Mol. Neurodegener..

[57] Samudra N., Lane-Donovan C., VandeVrede L., Boxer A.L. (2023). Tau pathology in neurodegenerative disease: Disease mechanisms and therapeutic avenues. J. Clin. Investig..

[58] Leak R.K., Clark R.N., Abbas M., Xu F., Brodsky J.L., Chen J., Hu X., Luk K.C. (2024). Current insights and assumptions on α-synuclein in Lewy body disease. Acta Neuropathol..

[59] Zhang W., Xiao D., Mao Q., Xia H. (2023). Role of neuroinflammation in neurodegeneration development. Signal Transduct. Target. Ther..

[60] Wu J., Wu J., Chen T., Cai J., Ren R. (2024). Protein aggregation and its affecting mechanisms in neurodegenerative diseases. Neurochem. Int..

[61] Chung C.G., Park S.S., Park J.H., Lee S.B. (2020). Dysregulated Plasma Membrane Turnover Underlying Dendritic Pathology in Neurodegenerative Diseases. Front. Cell. Neurosci..

[62] Lin M.-M., Liu N., Qin Z.-H., Wang Y. (2022). Mitochondrial-derived damage-associated molecular patterns amplify neuroinflammation in neurodegenerative diseases. Acta Pharmacol. Sin..

[63] Hivare P., Mujmer K., Swarup G., Gupta S., Bhatia D. (2023). Endocytic pathways of pathogenic protein aggregates in neurodegenerative diseases. Traffic.

[64] Klemmensen M.M., Borrowman S.H., Pearce C., Pyles B., Chandra B. (2024). Mitochondrial dysfunction in neurodegenerative disorders. Neurotherapeutics.

[65] Kwon O.-C., Song J.-J., Yang Y., Kim S.-H., Kim J.Y., Seok M.-J., Hwang I., Yu J.-W., Karmacharya J., Maeng H.-J. (2021). SGK1 inhibition in glia ameliorates pathologies and symptoms in Parkinson disease animal models. EMBO Mol. Med..

[66] Calì C., Cantando I., Veloz Castillo M.F., Gonzalez L., Bezzi P. (2024). Metabolic Reprogramming of Astrocytes in Pathological Conditions: Implications for Neurodegenerative Diseases. Int. J. Mol. Sci..

[67] Jack C.R., Bennett D.A., Blennow K., Carrillo M.C., Dunn B., Haeberlein S.B., Holtzman D.M., Jagust W., Jessen F., Karlawish J. (2018). NIA-AA Research Framework: Toward a biological definition of Alzheimer’s disease. Alzheimer’s Dement..

[68] Postuma R.B., Berg D., Stern M., Poewe W., Olanow C.W., Oertel W., Obeso J., Marek K., Litvan I., Lang A.E. (2015). MDS clinical diagnostic criteria for Parkinson’s disease. Mov. Disord..

[69] Morris H.R., Spillantini M.G., Sue C.M., Williams-Gray C.H. (2024). The pathogenesis of Parkinson’s disease. Lancet.

[70] Tabrizi S.J., Flower M.D., Ross C.A., Wild E.J. (2020). Huntington disease: New insights into molecular pathogenesis and therapeutic opportunities. Nat. Rev. Neurol..

[71] Donaldson J., Hensman Moss D., Ciosi M., Usdin K., Balmus G., Monckton D.G., Tabrizi S.J. (2026). Huntington disease: Somatic expansion, pathobiology and therapeutics. Nat. Rev. Neurol..

[72] Hayes L.R., Kalab P. (2022). Emerging Therapies and Novel Targets for TDP-43 Proteinopathy in ALS/FTD. Neurotherapeutics.

[73] Mead R.J., Shan N., Reiser H.J., Marshall F., Shaw P.J. (2023). Amyotrophic lateral sclerosis: A neurodegenerative disorder poised for successful therapeutic translation. Nat. Rev. Drug Discov..

[74] Boeve B.F., Boxer A.L., Kumfor F., Pijnenburg Y., Rohrer J.D. (2022). Advances and controversies in frontotemporal dementia: Diagnosis, biomarkers, and therapeutic considerations. Lancet Neurol..

[75] Grossman M., Seeley W.W., Boxer A.L., Hillis A.E., Knopman D.S., Ljubenov P.A., Miller B., Piguet O., Rademakers R., Whitwell J.L. (2023). Frontotemporal lobar degeneration. Nat. Rev. Dis. Primers.

[76] Yamada M., Komatsu J., Nakamura K., Sakai K., Samuraki-Yokohama M., Nakajima K., Yoshita M. (2020). Diagnostic Criteria for Dementia with Lewy Bodies: Updates and Future Directions. J. Mov. Disord..

[77] Noguchi-Shinohara M., Ono K. (2023). The Mechanisms of the Roles of α-synuclein, Amyloid-β, and Tau Protein in the Lewy Body Diseases: Pathogenesis, Early Detection, and Therapeutics. Int. J. Mol. Sci..

[78] Zarkali A., Bartl M., Fox N.C., Tan L.C.S., Mollenhauer B., Weil R.S. (2025). Diagnostic and other biomarkers of dementia with Lewy bodies: From research to clinical settings. Lancet Neurol..

[79] Krismer F., Fanciulli A., Meissner W.G., Coon E.A., Wenning G.K. (2024). Multiple system atrophy: Advances in pathophysiology, diagnosis, and treatment. Lancet Neurol..

[80] Liu M., Wang Z., Shang H. (2024). Multiple system atrophy: An update and emerging directions of biomarkers and clinical trials. J. Neurol..

[81] Chen Z., Mei L., Huang Y. (2026). Multiple system atrophy: Advances in pathogenesis and emerging therapeutic strategies. J. Neurol..

[82] Höglinger G.U., Respondek G., Stamelou M., Kurz C., Josephs K.A., Lang A.E., Mollenhauer B., Müller U., Nilsson C., Whitwell J.L. (2017). Clinical diagnosis of progressive supranuclear palsy: The movement disorder society criteria. Mov. Disord..

[83] Rowe J.B., Holland N., Rittman T. (2021). Progressive supranuclear palsy: Diagnosis and management. Pract. Neurol..

[84] Currens L., Pantelyat A. (2024). Progressive Supranuclear Palsy Diagnosis and Treatment. Curr. Treat. Options Neurol..

[85] Kovacs G.G. (2015). Invited review: Neuropathology of tauopathies: Principles and practice. Neuropathol. Appl. Neurobiol..

[86] Constantinides V.C., Paraskevas G.P., Paraskevas P.G., Stefanis L., Kapaki E. (2019). Corticobasal degeneration and corticobasal syndrome: A review. Clin. Park. Relat. Disord..

[87] Heemels M.-T. (2016). Neurodegenerative diseases. Nature.

[88] Yang H.-M. (2025). Overcoming the Blood-Brain Barrier: Advanced Strategies in Targeted Drug Delivery for Neurodegenerative Diseases. Pharmaceutics.

[89] Mistretta M., Farini A., Torrente Y., Villa C. (2023). Multifaceted nanoparticles: Emerging mechanisms and therapies in neurodegenerative diseases. Brain.

[90] Genchi G., Lauria G., Catalano A., Carocci A., Sinicropi M.S. (2024). Neuroprotective Effects of Curcumin in Neurodegenerative Diseases. Foods.

[91] Liu J., Wang T., Dong J., Lu Y. (2025). The blood-brain barriers: Novel nanocarriers for central nervous system diseases. J. Nanobiotechnol..

[92] Meng F., Wang J., Ping Q., Yeo Y. (2018). Quantitative Assessment of Nanoparticle Biodistribution by Fluorescence Imaging, Revisited. ACS Nano.

[93] Sierri G., Patrucco M., Ferrario D., Renda A., Comi S., Ciprandi M., Fontanini V., Sica F.S., Sesana S., Costa Verdugo M. (2024). Targeting specific brain districts for advanced nanotherapies: A review from the perspective of precision nanomedicine. Wiley Interdiscip. Rev. Nanomed. Nanobiotechnol..

[94] Katsoulaki E.-E., Dimopoulos D., Hadjipavlou-Litina D. (2025). Multitarget Compounds Designed for Alzheimer, Parkinson, and Huntington Neurodegeneration Diseases. Pharmaceuticals.

[95] Nagai M., Koebis M., Sasaki K., Kobayashi C., Daidoji K., Ishida T. (2025). Persistence and adherence to levodopa adjunct medications in elderly patients with Parkinson’s disease: A retrospective cohort study using a Japanese claims database. Front. Neurol..

[96] Mehta N.V., Kapadia A., Khambete M., Abhyankar A. (2025). The Evolution of AChE Inhibitors in Alzheimer’s Disease: From Single-Target to Multi-Target Ligands. ChemBioChem.

[97] Chen J.-C., Li L.-M., Gao J.-Q. (2019). Biomaterials for local drug delivery in central nervous system. Int. J. Pharm..

[98] Teixeira M.I., Lopes C.M., Amaral M.H., Costa P.C. (2024). Navigating Neurotoxicity and Safety Assessment of Nanocarriers for Brain Delivery: Strategies and Insights. Acta Biomater..

[99] Anwar M.M., Özkan E., Gürsoy-Özdemir Y. (2022). The role of extracellular matrix alterations in mediating astrocyte damage and pericyte dysfunction in Alzheimer’s disease: A comprehensive review. Eur. J. Neurosci..

[100] Shen K., Shi Y., Wang X., Leung S.W. (2024). Cellular Components of the Blood-Brain Barrier and Their Involvement in Aging-Associated Cognitive Impairment. Aging Dis..

[101] Ayloo S., Gu C. (2019). Transcytosis at the blood-brain barrier. Curr. Opin. Neurobiol..

[102] Wu D., Chen Q., Chen X., Han F., Chen Z., Wang Y. (2023). The blood-brain barrier: Structure, regulation, and drug delivery. Signal Transduct. Target. Ther..

[103] Yue Q., Hoi M.P.M. (2023). Emerging roles of astrocytes in blood-brain barrier disruption upon amyloid-beta insults in Alzheimer’s disease. Neural Regen. Res..

[104] Tsintzou A., Poirier R., Harati R., Kustubayeva A., Disdier C., Hamoudi R., Mabondzo A. (2025). Bridging regional neurovascular unit heterogeneity and cognitive function: A review. Fluids Barriers CNS.

[105] Chen T., Dai Y., Hu C., Lin Z., Wang S., Yang J., Zeng L., Li S., Li W. (2024). Cellular and molecular mechanisms of the blood-brain barrier dysfunction in neurodegenerative diseases. Fluids Barriers CNS.

[106] Takata F., Nakagawa S., Matsumoto J., Dohgu S. (2021). Blood-Brain Barrier Dysfunction Amplifies the Development of Neuroinflammation: Understanding of Cellular Events in Brain Microvascular Endothelial Cells for Prevention and Treatment of BBB Dysfunction. Front. Cell. Neurosci..

[107] Kadry H., Noorani B., Cucullo L. (2020). A blood-brain barrier overview on structure, function, impairment, and biomarkers of integrity. Fluids Barriers CNS.

[108] Wang Q., Huang X., Su Y., Yin G., Wang S., Yu B., Li H., Qi J., Chen H., Zeng W. (2022). Activation of Wnt/β-catenin pathway mitigates blood-brain barrier dysfunction in Alzheimer’s disease. Brain.

[109] Thomsen M.S., Humle N., Hede E., Moos T., Burkhart A., Thomsen L.B. (2021). The blood-brain barrier studied in vitro across species. PLoS ONE.

[110] Bhalerao A., Sivandzade F., Archie S.R., Chowdhury E.A., Noorani B., Cucullo L. (2020). In vitro modeling of the neurovascular unit: Advances in the field. Fluids Barriers CNS.

[111] Uchida Y., Ohtsuki S., Katsukura Y., Ikeda C., Suzuki T., Kamiie J., Terasaki T. (2011). Quantitative targeted absolute proteomics of human blood-brain barrier transporters and receptors. J. Neurochem..

[112] Yokel R.A. (2020). Nanoparticle brain delivery: A guide to verification methods. Nanomedicine.

[113] Wehn A.C., Krestel E., Harapan B.N., Klymchenko A., Plesnila N., Khalin I. (2024). To see or not to see: In vivo nanocarrier detection methods in the brain and their challenges. J. Control. Release.

[114] Farshbaf M., Davaran S., Rahimi F., Annabi N., Salehi R., Akbarzadeh A. (2018). Carbon quantum dots: Recent progresses on synthesis, surface modification and applications. Artif. Cells Nanomed. Biotechnol..

[115] Wu J., Liu J., Liu X., Zheng J., Chen L., Yang Y., Su C. (2024). Carbon Dots and Their Films with Narrow Full Width at Half Maximum Orange Emission. Molecules.

[116] Sun L., Zhao Y., Peng H., Zhou J., Zhang Q., Yan J., Liu Y., Guo S., Wu X., Li B. (2024). Carbon dots as a novel photosensitizer for photodynamic therapy of cancer and bacterial infectious diseases: Recent advances. J. Nanobiotechnol..

[117] Wu T., Bai X., Zhang Y., Dai E., Ma J., Yu C., He C., Li Q., Yang Y., Kong H. (2024). Natural medicines-derived carbon dots as novel oral antioxidant administration strategy for ulcerative colitis therapy. J. Nanobiotechnol..

[118] Chen B.B., Liu M.L., Huang C.Z. (2021). Recent advances of carbon dots in imaging-guided theranostics. TrAC Trends Anal. Chem..

[119] Panico S., Capolla S., Bozzer S., Toffoli G., Dal Bo M., Macor P. (2022). Biological Features of Nanoparticles: Protein Corona Formation and Interaction with the Immune System. Pharmaceutics.

[120] Wang H., Ai L., Song Z., Nie M., Xiao J., Li G., Lu S. (2023). Surface Modification Functionalized Carbon Dots. Chem. Eur. J..

[121] Reagen S., Wu Y., Shahni R., Sun W., Zhang J., Chu Q.R., Hou X., Combs C., Zhao J.X. (2022). Development of Red-Emissive Porphyrin Graphene Quantum Dots (PGQDs) for Biological Cell-Labeling Applications. ACS Omega.

[122] Dehvari K., Chiu S.-H., Lin J.-S., Girma W.M., Ling Y.-C., Chang J.-Y. (2020). Heteroatom doped carbon dots with nanoenzyme like properties as theranostic platforms for free radical scavenging, imaging, and chemotherapy. Acta Biomater..

[123] Yang D., Li L., Cao L., Chang Z., Mei Q., Yan R., Ge M., Jiang C., Dong W.-F. (2020). Green Synthesis of Lutein-Based Carbon Dots Applied for Free-Radical Scavenging within Cells. Materials.

[124] Zhou Y., Zhang W., Leblanc R.M. (2022). Structure-Property-Activity Relationships in Carbon Dots. J. Phys. Chem. B.

[125] Innocenzi P., Stagi L. (2023). Carbon dots as oxidant-antioxidant nanomaterials, understanding the structure-properties relationship. A critical review. Nano Today.

[126] Manikandan V., Lee N.Y. (2022). Green synthesis of carbon quantum dots and their environmental applications. Environ. Res..

[127] Liu Y., Zhang L., Cai H., Qu X., Chang J., Waterhouse G.I.N., Lu S. (2024). Biomass-derived carbon dots with pharmacological activity for biomedicine: Recent advances and future perspectives. Sci. Bull..

[128] Xu J., Huang B.-B., Lai C.-M., Lu Y.-S., Shao J.-W. (2024). Advancements in the synthesis of carbon dots and their application in biomedicine. J. Photochem. Photobiol. B.

[129] Xiong Y., Schneider J., Ushakova E.V., Rogach A.L. (2018). Influence of molecular fluorophores on the research field of chemically synthesized carbon dots. Nano Today.

[130] Sun S., Yu Y., Jo Y., Han J.H., Xue Y., Cho M., Bae S.-J., Ryu D., Park W., Ha K.-T. (2025). Impact of extraction techniques on phytochemical composition and bioactivity of natural product mixtures. Front. Pharmacol..

[131] Balekundri A., Mannur V. (2020). Quality control of the traditional herbs and herbal products: A review. Future J. Pharm. Sci..

[132] Mansuriya B.D., Altintas Z. (2021). Carbon Dots: Classification, Properties, Synthesis, Characterization, and Applications in Health Care-An Updated Review (2018–2021). Nanomaterials.

[133] Sharma A., Das J. (2019). Small molecules derived carbon dots: Synthesis and applications in sensing, catalysis, imaging, and biomedicine. J. Nanobiotechnol..

[134] Zhu X., Li C., Yan K., Hao Y., Chen S., Zhou Y., Xu M. (2026). Baicalein-conjugated carbon dots as photoluminescent nanoprobes for enhanced antioxidant activity, bioimaging, and selective Fe3+ sensing. J. Photochem. Photobiol. A.

[135] Medina-Berríos N., Pantoja-Romero W., Lavín Flores A., Díaz Vélez S.C., Martínez Guadalupe A.C., Torres Mulero M.T., Kisslinger K., Martínez-Ferrer M., Morell G., Weiner B.R. (2024). Synthesis and Characterization of Carbon-Based Quantum Dots and Doped Derivatives for Improved Andrographolide’s Hydrophilicity in Drug Delivery Platforms. ACS Omega.

[136] Peng Z., Han X., Li S., Al-Youbi A.O., Bashammakh A.S., El-Shahawi M.S., Leblanc R.M. (2017). Carbon dots: Biomacromolecule interaction, bioimaging and nanomedicine. Coord. Chem. Rev..

[137] Yao Y., Zhou W., Cai K., Wen J., Zhang X. (2024). Advances in the study of the biological activity of polysaccharide-based carbon dots: A review. Int. J. Biol. Macromol..

[138] Chen J., Wang Q., Zhou J., Deng W., Yu Q., Cao X., Wang J., Shao F., Li Y., Ma P. (2017). Porphyra polysaccharide-derived carbon dots for non-viral co-delivery of different gene combinations and neuronal differentiation of ectodermal mesenchymal stem cells. Nanoscale.

[139] Maheshwari S., Singh A., Verma A. (2026). Polysaccharide-Based Carbon Quantum Dots: Synthesis and Theranostic Applications—A Review. Biomed. Mater. Devices.

[140] Strickland S.A., Fourroux L.A., McKay M.M., Pappas D. (2025). Protein-Derived Carbon Dots: Effects of Synthesis Parameters on Retained Protein Function. Adv. Ther..

[141] Ghataty D.S., Amer R.I., Amer M.A., Abdel Rahman M.F., Shamma R.N. (2023). Green Synthesis of Highly Fluorescent Carbon Dots from Bovine Serum Albumin for Linezolid Drug Delivery as Potential Wound Healing Biomaterial: Bio-Synergistic Approach, Antibacterial Activity, and In Vitro and Ex Vivo Evaluation. Pharmaceutics.

[142] Perumal S., Atchudan R., Edison T.N.J.I., Lee Y.R. (2021). Sustainable synthesis of multifunctional carbon dots using biomass and their applications: A mini-review. J. Environ. Chem. Eng..

[143] Surendran P., Lakshmanan A., Priya S.S., Balakrishnan K., Rameshkumar P., Kannan K., Geetha P., Hegde T.A., Vinitha G. (2020). Bioinspired fluorescence carbon quantum dots extracted from natural honey: Efficient material for photonic and antibacterial applications. Nano-Struct. Nano-Objects.

[144] Rodríguez-Varillas S., Fontanil T., Obaya Á.J., Fernández-González A., Murru C., Badía-Laíño R. (2022). Biocompatibility and Antioxidant Capabilities of Carbon Dots Obtained from Tomato (Solanum lycopersicum). Appl. Sci..

[145] Zhang J., Zou L., Li Q., Wu H., Sun Z., Xu X., Shi L., Sun Z., Ma G. (2023). Carbon Dots Derived from Traditional Chinese Medicines with Bioactivities: A Rising Star in Clinical Treatment. ACS Appl. Bio Mater..

[146] Li D., Xu K.-Y., Zhao W.-P., Liu M.-F., Feng R., Li D.-Q., Bai J., Du W.-L. (2022). Chinese Medicinal Herb-Derived Carbon Dots for Common Diseases: Efficacies and Potential Mechanisms. Front. Pharmacol..

[147] Li Y., Li W., Yang X., Kang Y., Zhang H., Liu Y., Lei B. (2021). *Salvia Miltiorrhiza*-Derived Carbon Dots as Scavengers of Reactive Oxygen Species for Reducing Oxidative Damage of Plants. ACS Appl. Nano Mater..

[148] Guo Z., Wang Z., Liu Y., Wu H., Zhang Q., Han J., Liu J., Zhang C. (2023). Carbon Dots from *Lycium barbarum* Attenuate Radiation-Induced Bone Injury by Inhibiting Senescence via METTL3/Clip3 in an m6A-Dependent Manner. ACS Appl. Mater. Interfaces.

[149] Zhang Y., Ma J., Zou P., Xia M., Wang S., Jin B., Lu Z., Zhai C., Huang H., Zhang Y. (2026). Natural drug-derived carbon dots as a novel oral dosing strategy for gouty arthritis. Int. Immunopharmacol..

[150] Zhou G., Shen X., Zhao Z., Yan R., Huang P., Tan R., Liang S., Zhi Y., Li J. (2021). Clinical Applications, Active Components and Mechanisms of Haemostatic Effects of Charred Chinese Medicines. J. Dis. Med. Plants.

[151] Luo J., Zhang M., Cheng J., Wu S., Xiong W., Kong H., Zhao Y., Qu H. (2018). Hemostatic effect of novel carbon dots derived from *Cirsium setosum* Carbonisata. RSC Adv..

[152] Zhang Z., Hu W., Yu A., Kuang H., Wang M. (2024). Hemostatic bioactivity and mechanism of novel *Rubia cordifolia* L.-derived carbon dots. Nanoscale Adv..

[153] Lv J., Tian H., Pan L., Chen Z., Li M., Ghiladi R.A., Qin Z., Yin X. (2024). Biomass derived carbon dots with antibacterial and anti-inflammatory properties for the treatment of wound healing. Chem. Eng. Sci..

[154] Tejwan N., Kundu M., Ghosh N., Chatterjee S., Sharma A., Singh T.A., Das J., Sil P.C. (2022). Synthesis of green carbon dots as bioimaging agent and drug delivery system for enhanced antioxidant and antibacterial efficacy. Inorg. Chem. Commun..

[155] Han B., Shen L., Xie H., Huang Q., Zhao D., Huang X., Chen X., Li J. (2023). Synthesis of Carbon Dots with Hemostatic Effects Using Traditional Chinese Medicine as a Biomass Carbon Source. ACS Omega.

[156] Lu F., Ma Y., Wang H., Zhang M., Wang B., Zhang Y., Huang H., Liao F., Liu Y., Kang Z. (2021). Water-solvable carbon dots derived from curcumin and citric acid with enhanced broad-spectrum antibacterial and antibiofilm activity. Mater. Today Commun..

[157] Li S., Peng Z., Dallman J., Baker J., Othman A.M., Blackwelder P.L., Leblanc R.M. (2016). Crossing the blood-brain-barrier with transferrin conjugated carbon dots: A zebrafish model study. Colloids Surf. B.

[158] Wang Y., Hu A. (2014). Carbon quantum dots: Synthesis, properties and applications. J. Mater. Chem. C.

[159] Seven E.S., Seven Y.B., Zhou Y., Poudel-Sharma S., Diaz-Rucco J.J., Kirbas Cilingir E., Mitchell G.S., Van Dyken J.D., Leblanc R.M. (2021). Crossing the blood-brain barrier with carbon dots: Uptake mechanism and in vivo cargo delivery. Nanoscale Adv..

[160] Seven Y.B., Seven E.S., Kirbas Cilingir E., Parikh K., Aydin M., Luca E.K., Nair J., Leblanc R.M. (2025). Rapid mapping of spinal and supraspinal connectome via self-targeting glucose-based carbon dots. Nanoscale.

[161] Mintz K.J., Mercado G., Zhou Y., Ji Y., Hettiarachchi S.D., Liyanage P.Y., Pandey R.R., Chusuei C.C., Dallman J., Leblanc R.M. (2019). Tryptophan carbon dots and their ability to cross the blood-brain barrier. Colloids Surf. B.

[162] Guo W., Ji M., Li Y., Qian M., Qin Y., Li W., Nie H., Lv W., Jiang G., Huang R. (2024). Iron ions-sequestrable and antioxidative carbon dot-based nano-formulation with nitric oxide release for Parkinson’s disease treatment. Biomaterials.

[163] Zhou Y., Peng Z., Seven E.S., Leblanc R.M. (2018). Crossing the blood-brain barrier with nanoparticles. J. Control. Release.

[164] Xiao G., Gan L.-S. (2013). Receptor-mediated endocytosis and brain delivery of therapeutic biologics. Int. J. Cell Biol..

[165] Zhai L., Gao Y., Yang H., Wang H., Liao B., Cheng Y., Liu C., Che J., Xia K., Zhang L. (2025). A ROS-Responsive nanoparticle for nuclear gene delivery and autophagy restoration in Parkinson’s disease therapy. Biomaterials.

[166] Luo W., Zhang L., Li X., Zheng J., Chen Q., Yang Z., Cheng M., Chen Y., Wu Y., Zhang W. (2022). Green functional carbon dots derived from herbal medicine ameliorate blood—Brain barrier permeability following traumatic brain injury. Nano Res..

[167] Sulhan S., Lyon K.A., Shapiro L.A., Huang J.H. (2020). Neuroinflammation and blood-brain barrier disruption following traumatic brain injury: Pathophysiology and potential therapeutic targets. J. Neurosci. Res..

[168] Turgutalp B., Kizil C. (2024). Multi-target drugs for Alzheimer’s disease. Trends Pharmacol. Sci..

[169] Girma W.M., Demissie G.G., Mekuria S.L., Kizhepat S., Subrahmanya T.M., Rasal A.S., Berhe B.A., Gedda G., Park Y.-J., Chang J.-Y. (2026). Carbon dots penetrating the blood-brain barrier for central nervous system nanomedicine. Theranostics.

[170] Manoharan S., Guillemin G.J., Abiramasundari R.S., Essa M.M., Akbar M., Akbar M.D. (2016). The Role of Reactive Oxygen Species in the Pathogenesis of Alzheimer’s Disease, Parkinson’s Disease, and Huntington’s Disease: A Mini Review. Oxid. Med. Cell. Longev..

[171] Yu Z., Luo F. (2024). The Role of Reactive Oxygen Species in Alzheimer’s Disease: From Mechanism to Biomaterials Therapy. Adv. Healthc. Mater..

[172] Houldsworth A. (2024). Role of oxidative stress in neurodegenerative disorders: A review of reactive oxygen species and prevention by antioxidants. Brain Commun..

[173] Liu Z.J., Roy A., Zheng Y., Annabi N. (2025). Harnessing carbon nanomaterials for reactive oxygen species regulation: Insights into generation, scavenging, and sensing. Adv. Drug Deliv. Rev..

[174] Fan T., Cao X., Wang C., Shao X., Wang X., Guan P., Hu X. (2025). Vitamin C derived carbon dots: Inhibiting amyloid aggregation and scavenging reactive oxygen species. New J. Chem..

[175] Kuznietsova H., Géloën A., Dziubenko N., Zaderko A., Alekseev S., Lysenko V., Skryshevsky V. (2023). In vitro and in vivo toxicity of carbon dots with different chemical compositions. Discov. Nano.

[176] Wen P., Sun Z., Gou F., Wang J., Fan Q., Zhao D., Yang L. (2025). Oxidative stress and mitochondrial impairment: Key drivers in neurodegenerative disorders. Ageing Res. Rev..

[177] Uttara B., Singh A.V., Zamboni P., Mahajan R.T. (2009). Oxidative stress and neurodegenerative diseases: A review of upstream and downstream antioxidant therapeutic options. Curr. Neuropharmacol..

[178] Angelova P.R., Esteras N., Abramov A.Y. (2021). Mitochondria and lipid peroxidation in the mechanism of neurodegeneration: Finding ways for prevention. Med. Res. Rev..

[179] Abramov A.Y., Potapova E.V., Dremin V.V., Dunaev A.V. (2020). Interaction of Oxidative Stress and Misfolded Proteins in the Mechanism of Neurodegeneration. Life.

[180] Zhang S., Tao H., Zheng X., Zhao S., Wei Y. (2025). Multifunctional carbon dots from *Polygonatum kingianum* and *Gastrodia elata*: Synthesis, characterization, and biomedical applications. RSC Adv..

[181] Kumar J., Delgado S.A., Sarma H., Narayan M. (2023). Caffeic acid recarbonization: A green chemistry, sustainable carbon nano material platform to intervene in neurodegeneration induced by emerging contaminants. Environ. Res..

[182] Lin X., Liu W., Dong X., Sun Y. (2023). Epigallocatechin gallate-derived carbonized polymer dots: A multifunctional scavenger targeting Alzheimer’s β-amyloid plaques. Acta Biomater..

[183] Gao C., Jiang J., Tan Y., Chen S. (2023). Microglia in neurodegenerative diseases: Mechanism and potential therapeutic targets. Signal Transduct. Target. Ther..

[184] Biswas K. (2023). Microglia mediated neuroinflammation in neurodegenerative diseases: A review on the cell signaling pathways involved in microglial activation. J. Neuroimmunol..

[185] Huang Y., Li M., Dong L., He C., Zou P., Xia M., Jin B., Wang S., Lu Z., Qu H. (2025). Crinis Carbonisatus-Derived Carbon Dot Suspension Alleviates Temporal Lobe Epilepsy. Pharmaceuticals.

[186] Jiang J., Pan Z., Su Y., Dai L., Xu N., Wu H., Chen X. (2025). Carbon dots from purple sweet potato as a promising anti-inflammatory biomaterial for alleviating the LPS-induced inflammation in macrophages. J. Nanobiotechnol..

[187] Wei Z., Dong X., Sun Y. (2025). Design of Red Fluorescence Ce-Based Carbon Dots of High and Balanced Multiple Functions against Alzheimer’s β-amyloid Fibrillization. ACS Appl. Mater. Interfaces.

[188] Xiao M., Pan Y., Tang S., Guo T., Yang L., Chen G. (2025). Carbon quantum dots amplify beneficial effects of EGCG against neural injuries by NLRP3 inflammasome after intracerebral hemorrhage. Int. J. Pharm..

[189] Li K., Ling H., Wang X., Xie Q., Gu C., Luo W., Qiu P. (2024). The role of NF-κB signaling pathway in reactive astrocytes among neurodegeneration after methamphetamine exposure by integrated bioinformatics. Prog. Neuropsychopharmacol. Biol. Psychiatry.

[190] Su W.-S., Wu C.-H., Song W.-S., Chen S.-F., Yang F.-Y. (2023). Low-intensity pulsed ultrasound ameliorates glia-mediated inflammation and neuronal damage in experimental intracerebral hemorrhage conditions. J. Transl. Med..

[191] Bauernfeind F.G., Horvath G., Stutz A., Alnemri E.S., MacDonald K., Speert D., Fernandes-Alnemri T., Wu J., Monks B.G., Fitzgerald K.A. (2009). Cutting edge: NF-κB activating pattern recognition and cytokine receptors license NLRP3 inflammasome activation by regulating NLRP3 expression. J. Immunol..

[192] Liu T., Zhang L., Joo D., Sun S.-C. (2017). NF-κB signaling in inflammation. Signal Transduct. Target. Ther..

[193] Xu W., Huang Y., Zhou R. (2025). NLRP3 inflammasome in neuroinflammation and central nervous system diseases. Cell. Mol. Immunol..

[194] Soto C. (2003). Unfolding the role of protein misfolding in neurodegenerative diseases. Nat. Rev. Neurosci..

[195] Ciechanover A., Kwon Y.T. (2015). Degradation of misfolded proteins in neurodegenerative diseases: Therapeutic targets and strategies. Exp. Mol. Med..

[196] Sweeney P., Park H., Baumann M., Dunlop J., Frydman J., Kopito R., McCampbell A., Leblanc G., Venkateswaran A., Nurmi A. (2017). Protein misfolding in neurodegenerative diseases: Implications and strategies. Transl. Neurodegener..

[197] Wang J., Dai L., Zhang Z. (2025). Protein aggregation in neurodegenerative diseases. Chin. Med. J..

[198] Hu C., Lin M., Wang C., Zhang S. (2025). Current Understanding of Protein Aggregation in Neurodegenerative Diseases. Int. J. Mol. Sci..

[199] Zhang Y.P., Kedia S., Klenerman D. (2025). Rethinking neurodegeneration through a co-proteinopathy lens. Trends Neurosci..

[200] Yan C., Shao X., Wang Y., Tang S., Li S., Wang C., Bai M., Qi Y., Ma Y., Zhao R. (2025). Application of carbon dots-based nanomaterials in amyloid aggregation disease. Carbon.

[201] Rinauro D.J., Chiti F., Vendruscolo M., Limbocker R. (2024). Misfolded protein oligomers: Mechanisms of formation, cytotoxic effects, and pharmacological approaches against protein misfolding diseases. Mol. Neurodegener..

[202] Bashir S., Aiman A., Shahid M., Chaudhary A.A., Sami N., Basir S.F., Hassan I., Islam A. (2024). Amyloid-induced neurodegeneration: A comprehensive review through aggregomics perception of proteins in health and pathology. Ageing Res. Rev..

[203] Damian Guerrero E., Lopez-Velazquez A.M., Ahlawat J., Narayan M. (2021). Carbon Quantum Dots for Treatment of Amyloid Disorders. ACS Appl. Nano Mater..

[204] Wei Z., Dong X., Sun Y. (2024). Quercetin-derived red emission carbon dots: A multifunctional theranostic nano-agent against Alzheimer’s β-amyloid fibrillogenesis. Colloids Surf. B.

[205] Prabhu M.P.T., Chrungoo S., Sarkar N. (2023). Amine Group Surface-Functionalized Carbon Quantum Dots Exhibit Anti-amyloidogenic Effects Towards Hen Egg White Lysozyme by Inducing Formation of Nontoxic Spherical Aggregates. Protein J..

[206] Li D., Wang S., Dong J., Li J., Wang X., Liu F., Ba X. (2024). Inhibition and disaggregation effect of flavonoid-derived carbonized polymer dots on protein amyloid aggregation. Colloids Surf. B.

[207] Bathla M., Saini T.C., Teji N., Randhawa S., Bhardwaj R., Acharya A. (2026). Nanoenabled Sequestration of Redox Copper Modulates Lysozyme Aggregation and Maintains Lipid Homeostasis to Alleviate Mitochondrial Health. Small.

[208] Li L., Lu Y., Ye X., Zhang C., Liu J., Yang Z., Hao J. (2025). Hydrophobic Carbon Dots Prevent α-synucleinopathy and Suppress Neuroinflammation to Treat Parkinson’s Disease. Aggregate.

[209] Zhang W., Smith N., Zhou Y., McGee C.M., Bartoli M., Fu S., Chen J., Domena J.B., Joji A., Burr H. (2024). Carbon dots as dual inhibitors of tau and amyloid-beta aggregation for the treatment of Alzheimer’s disease. Acta Biomater..

[210] Kim D.J., Yoo J.M., Suh Y., Kim D., Kang I., Moon J., Park M., Kim J., Kang K.-S., Hong B.H. (2021). Graphene Quantum Dots from Carbonized Coffee Bean Wastes for Biomedical Applications. Nanomaterials.

[211] Kumar R., Vincy A., Rani K., Jain N., Singh S., Agarwal A., Vankayala R. (2023). Facile Synthesis of Multifunctional Carbon Dots Derived from Camel Milk for Mn7+ Sensing and Antiamyloid and Anticancer Activities. ACS Omega.

[212] Kochman U., Sitka H., Kuźniar J., Czaja M., Kozubek P., Beszłej J.A., Leszek J. (2026). Targeted Nanoparticles for Drug Delivery Across the Blood-Brain Barrier in Early and Late Stages of Alzheimer’s Disease: A Review. Mol. Neurobiol..

[213] Calabrese G., De Luca G., Nocito G., Rizzo M.G., Lombardo S.P., Chisari G., Forte S., Sciuto E.L., Conoci S. (2021). Carbon Dots: An Innovative Tool for Drug Delivery in Brain Tumors. Int. J. Mol. Sci..

[214] Wu J. (2021). The Enhanced Permeability and Retention (EPR) Effect: The Significance of the Concept and Methods to Enhance Its Application. J. Pers. Med..

[215] Haqqani A.S., Bélanger K., Stanimirovic D.B. (2024). Receptor-mediated transcytosis for brain delivery of therapeutics: Receptor classes and criteria. Front. Drug Deliv..

[216] Han M., Yi B., Song R., Wang D., Huang N., Ma Y., Zhao L., Liu S., Zhang H., Xu R. (2025). Fucoidan-derived carbon dots as nanopenetrants of blood-brain barrier for Parkinson’s disease treatment. J. Colloid Interface Sci..

[217] Gárate-Vélez L., Quintana M. (2025). Carbon-based nanomaterials: Interactions with cells, brain therapies, and neural sensing. J. Mater. Sci. Mater. Eng..

[218] Hu Y., Cui J., Sun J., Liu X., Gao S., Mei X., Wu C., Tian H. (2024). A novel biomimetic nanovesicle containing caffeic acid-coupled carbon quantum dots for the the treatment of Alzheimer’s disease via nasal administration. J. Nanobiotechnol..

[219] Kaurav H., Verma D., Bansal A., Kapoor D.N., Sheth S. (2023). Progress in drug delivery and diagnostic applications of carbon dots: A systematic review. Front. Chem..

[220] Mathew S.A., Praveena P., Dhanavel S., Manikandan R., Senthilkumar S., Stephen A. (2020). Luminescent chitosan/carbon dots as an effective nano-drug carrier for neurodegenerative diseases. RSC Adv..

[221] He X., Xie J., Zhang J., Wang X., Jia X., Yin H., Qiu Z., Yang Z., Chen J., Ji Z. (2022). Acid-Responsive Dual-Targeted Nanoparticles Encapsulated Aspirin Rescue the Immune Activation and Phenotype in Autism Spectrum Disorder. Adv. Sci..

[222] Yuan X., Jia Z., Li J., Liu Y., Huang Y., Gong Y., Guo X., Chen X., Cen J., Liu J. (2021). A diselenide bond-containing ROS-responsive ruthenium nanoplatform delivers nerve growth factor for Alzheimer’s disease management by repairing and promoting neuron regeneration. J. Mater. Chem. B.

[223] Gao L., Wang J., Bi Y. (2025). Nanotechnology for Neurodegenerative Diseases: Recent Progress in Brain-Targeted Delivery, Stimuli-Responsive Platforms, and Organelle-Specific Therapeutics. Int. J. Nanomed..

[224] Yang L., Yin T., Liu Y., Sun J., Zhou Y., Liu J. (2016). Gold nanoparticle-capped mesoporous silica-based H2O2-responsive controlled release system for Alzheimer’s disease treatment. Acta Biomater..

[225] Kuang Y., Zhang J., Xiong M., Zeng W., Lin X., Yi X., Luo Y., Yang M., Li F., Huang Q. (2020). A Novel Nanosystem Realizing Curcumin Delivery Based on Fe_3_O_4_@Carbon Dots Nanocomposite for Alzheimer’s Disease Therapy. Front. Bioeng. Biotechnol..

[226] Mathur P., Mori M., Patel F., Upadhyay K., Baxi D., Desai A. (2025). Curcuminoid-Linked Lemon-Derived Carbon Dots for pH-Triggered Drug Release. ACS Omega.

[227] Sinha T., Bokhari S.F.H., Khan M.U., Sarim Shaheer M., Amir M., Zia B.F., Bakht D., Javed M.A., Almadhoun M.K.I.K., Burhanuddin M. (2024). Gazing Beyond the Horizon: A Systematic Review Unveiling the Theranostic Potential of Quantum Dots in Alzheimer’s Disease. Cureus.

[228] Minami S.S., Sun B., Popat K., Kauppinen T., Pleiss M., Zhou Y., Ward M.E., Floreancig P., Mucke L., Desai T. (2012). Selective targeting of microglia by quantum dots. J. Neuroinflamm..

[229] Snipstad S., Hak S., Baghirov H., Sulheim E., Mørch Ý., Lélu S., von Haartman E., Bäck M., Nilsson K.P.R., Klymchenko A.S. (2017). Labeling nanoparticles: Dye leakage and altered cellular uptake. Cytom. Part A.

[230] Zhang Q., Wang R., Feng B., Zhong X., Ostrikov K.K. (2021). Photoluminescence mechanism of carbon dots: Triggering high-color-purity red fluorescence emission through edge amino protonation. Nat. Commun..

[231] Pan L., Sun S., Zhang L., Jiang K., Lin H. (2016). Near-infrared emissive carbon dots for two-photon fluorescence bioimaging. Nanoscale.

[232] Shi W., Li J., Pu J., Cheng G., Liu Y., Xiao S., Cao J. (2025). *Epigynum auritum*-Derived Near-Infrared Carbon Dots for Bioimaging and Antimicrobial Applications. Molecules.

[233] Li L., Zhang R., Lu C., Sun J., Wang L., Qu B., Li T., Liu Y., Li S. (2017). In situ synthesis of NIR-light emitting carbon dots derived from spinach for bio-imaging applications. J. Mater. Chem. B.

[234] Liu J., Kong T., Xiong H.-M. (2022). Mulberry-Leaves-Derived Red-Emissive Carbon Dots for Feeding Silkworms to Produce Brightly Fluorescent Silk. Adv. Mater..

[235] Ding H., Ji Y., Wei J.-S., Gao Q.-Y., Zhou Z.-Y., Xiong H.-M. (2017). Facile synthesis of red-emitting carbon dots from pulp-free lemon juice for bioimaging. J. Mater. Chem. B.

[236] Liu J., Geng Y., Li D., Yao H., Huo Z., Li Y., Zhang K., Zhu S., Wei H., Xu W. (2020). Deep Red Emissive Carbonized Polymer Dots with Unprecedented Narrow Full Width at Half Maximum. Adv. Mater..

[237] Bloch D.N., Ben Zichri S., Kolusheva S., Jelinek R. (2020). Tyrosine carbon dots inhibit fibrillation and toxicity of the human islet amyloid polypeptide. Nanoscale Adv..

[238] Luo W.-C., Jiang M., Li L., Bao L.-N., Yu X., Xu L. (2024). Deep eutectic solvent-assisted preparation of resveratrol-based carbon dots with high biocompatibility for in situ screening inhibitors on amyloid protein aggregation and scavenging reactive oxygen species. Sens. Actuators B.

[239] Guo F., Li Q., Zhang X., Liu Y., Jiang J., Cheng S., Yu S., Zhang X., Liu F., Li Y. (2022). Applications of Carbon Dots for the Treatment of Alzheimer’s Disease. Int. J. Nanomed..

[240] Helms H.C., Abbott N.J., Burek M., Cecchelli R., Couraud P.-O., Deli M.A., Förster C., Galla H.J., Romero I.A., Shusta E.V. (2016). In vitro models of the blood-brain barrier: An overview of commonly used brain endothelial cell culture models and guidelines for their use. J. Cereb. Blood Flow Metab..

[241] Katt M.E., Shusta E.V. (2020). In vitro models of the blood-brain barrier: Building in physiological complexity. Curr. Opin. Chem. Eng..

[242] Huang X., Zhang F., Zhu L., Choi K.Y., Guo N., Guo J., Tackett K., Anilkumar P., Liu G., Quan Q. (2013). Effect of injection routes on the biodistribution, clearance, and tumor uptake of carbon dots. ACS Nano.

[243] Wang P., Wang Y., Li P., Chen C., Ma S., Zhao L., He H., Yin T., Zhang Y., Tang X. (2023). Oral delivery of polyester nanoparticles for brain-targeting: Challenges and opportunities. Chin. Chem. Lett..

[244] Lochhead J.J., Davis T.P. (2019). Perivascular and Perineural Pathways Involved in Brain Delivery and Distribution of Drugs after Intranasal Administration. Pharmaceutics.

[245] Keller L.-A., Merkel O., Popp A. (2022). Intranasal drug delivery: Opportunities and toxicologic challenges during drug development. Drug Deliv. Transl. Res..

[246] Lochhead J.J., Thorne R.G. (2012). Intranasal delivery of biologics to the central nervous system. Adv. Drug Deliv. Rev..

[247] van Rooy I., Cakir-Tascioglu S., Hennink W.E., Storm G., Schiffelers R.M., Mastrobattista E. (2011). In vivo methods to study uptake of nanoparticles into the brain. Pharm. Res..

[248] Loryan I., Reichel A., Feng B., Bundgaard C., Shaffer C., Kalvass C., Bednarczyk D., Morrison D., Lesuisse D., Hoppe E. (2022). Unbound Brain-to-Plasma Partition Coefficient, Kp,uu,brain—A Game Changing Parameter for CNS Drug Discovery and Development. Pharm. Res..

[249] Liu N., Shi Y., Guo J., Li H., Wang Q., Song M., Shi Z., He L., Su X., Xie J. (2019). Radioiodinated tyrosine based carbon dots with efficient renal clearance for single photon emission computed tomography of tumor. Nano Res..

[250] Jiang Q., Liu L., Li Q., Cao Y., Chen D., Du Q., Yang X., Huang D., Pei R., Chen X. (2021). NIR-laser-triggered gadolinium-doped carbon dots for magnetic resonance imaging, drug delivery and combined photothermal chemotherapy for triple negative breast cancer. J. Nanobiotechnol..

[251] Wysocka A., Waluda Ł., Konefał R., Kasprzyk W. (2024). Liquid chromatography methods as a solution to inaccuracies associated with purity assessment of citric acid-based carbon dots. Microchem. J..

[252] Li Y., Bai G., Zeng S., Hao J. (2019). Theranostic Carbon Dots with Innovative NIR-II Emission for in Vivo Renal-Excreted Optical Imaging and Photothermal Therapy. ACS Appl. Mater. Interfaces.

[253] Choi H.S., Liu W., Misra P., Tanaka E., Zimmer J.P., Itty Ipe B., Bawendi M.G., Frangioni J.V. (2007). Renal clearance of quantum dots. Nat. Biotechnol..

[254] Liao J., Yao Y., Lee C.-H., Wu Y., Li P. (2021). In Vivo Biodistribution, Clearance, and Biocompatibility of Multiple Carbon Dots Containing Nanoparticles for Biomedical Application. Pharmaceutics.

[255] Boyes W.K., van Thriel C. (2020). Neurotoxicology of Nanomaterials. Chem. Res. Toxicol..

[256] Karmakar A., Zhang Q., Zhang Y. (2014). Neurotoxicity of nanoscale materials. J. Food Drug Anal..

[257] Essner J.B., Kist J.A., Polo-Parada L., Baker G.A. (2018). Artifacts and Errors Associated with the Ubiquitous Presence of Fluorescent Impurities in Carbon Nanodots. Chem. Mater..

[258] Hu Y., Seivert O., Tang Y., Karahan H.E., Bianco A. (2024). Carbon Dot Synthesis and Purification: Trends, Challenges and Recommendations. Angew. Chem. Int. Ed..

[259] Dobrovolskaia M.A. (2015). Pre-clinical immunotoxicity studies of nanotechnology-formulated drugs: Challenges, considerations and strategy. J. Control. Release.

[260] de la Harpe K.M., Kondiah P.P.D., Choonara Y.E., Marimuthu T., du Toit L.C., Pillay V. (2019). The Hemocompatibility of Nanoparticles: A Review of Cell-Nanoparticle Interactions and Hemostasis. Cells.

[261] La-Beck N.M., Islam M.R., Markiewski M.M. (2020). Nanoparticle-Induced Complement Activation: Implications for Cancer Nanomedicine. Front. Immunol..

[262] Ozyurt D., Kobaisi M.A., Hocking R.K., Fox B. (2023). Properties, synthesis, and applications of carbon dots: A review. Carbon Trends.

[263] Cai D., Zhong X., Xu L., Xiong Y., Deng W., Zou G., Hou H., Ji X. (2025). Biomass-derived carbon dots: Synthesis, modification and application in batteries. Chem. Sci..

[264] Faria M., Björnmalm M., Thurecht K.J., Kent S.J., Parton R.G., Kavallaris M., Johnston A.P.R., Gooding J.J., Corrie S.R., Boyd B.J. (2018). Minimum information reporting in bio-nano experimental literature. Nat. Nanotechnol..

[265] Caputo F., Clogston J., Calzolai L., Rösslein M., Prina-Mello A. (2019). Measuring particle size distribution of nanoparticle enabled medicinal products, the joint view of EUNCL and NCI-NCL. A step by step approach combining orthogonal measurements with increasing complexity. J. Control. Release.

[266] Lin P.-C., Lin S., Wang P.C., Sridhar R. (2014). Techniques for physicochemical characterization of nanomaterials. Biotechnol. Adv..

[267] O’Brown N.M., Pfau S.J., Gu C. (2018). Bridging barriers: A comparative look at the blood-brain barrier across organisms. Genes Dev..

[268] Song H.W., Foreman K.L., Gastfriend B.D., Kuo J.S., Palecek S.P., Shusta E.V. (2020). Transcriptomic comparison of human and mouse brain microvessels. Sci. Rep..

[269] Zhang W., Liu Q.Y., Haqqani A.S., Leclerc S., Liu Z., Fauteux F., Baumann E., Delaney C.E., Ly D., Star A.T. (2020). Differential expression of receptors mediating receptor-mediated transcytosis (RMT) in brain microvessels, brain parenchyma and peripheral tissues of the mouse and the human. Fluids Barriers CNS.

[270] Knox E.G., Aburto M.R., Clarke G., Cryan J.F., O’Driscoll C.M. (2022). The blood-brain barrier in aging and neurodegeneration. Mol. Psychiatry.

[271] Andjelkovic A.V., Situ M., Citalan-Madrid A.F., Stamatovic S.M., Xiang J., Keep R.F. (2023). Blood-Brain Barrier Dysfunction in Normal Aging and Neurodegeneration: Mechanisms, Impact, and Treatments. Stroke.

[272] Wevers N.R., De Vries H.E. (2023). Microfluidic models of the neurovascular unit: A translational view. Fluids Barriers CNS.

[273] Rizzuti M., Melzi V., Brambilla L., Quetti L., Sali L., Ottoboni L., Meneri M., Ratti A., Verde F., Ticozzi N. (2024). Shaping the Neurovascular Unit Exploiting Human Brain Organoids. Mol. Neurobiol..

[274] González-Gallego J., Todorov-Völgyi K., Müller S.A., Antesberger S., Todorov M.I., Malik R., Grimalt-Mirada R., Gonçalves C.C., Schifferer M., Kislinger G. (2026). A fully iPS-cell-derived 3D model of the human blood-brain barrier for exploring neurovascular disease mechanisms and therapeutic interventions. Nat. Neurosci..

[275] (2022). Drug Products, Including Biological Products, that Contain Nanomaterials: Guidance for Industry.

[276] (2022). Principles of Premarket Pathways for Combination Products: Guidance for Industry and FDA Staff.

[277] (2009). Pharmaceutical Development.

[278] (2008). Pharmaceutical Quality System.

[279] (2009). Guidance on Nonclinical Safety Studies for the Conduct of Human Clinical Trials and Marketing Authorization for Pharmaceuticals.

